# Developments in Synthetic Application of Selenium(IV) Oxide and Organoselenium Compounds as Oxygen Donors and Oxygen-Transfer Agents

**DOI:** 10.3390/molecules200610205

**Published:** 2015-06-03

**Authors:** Jacek Młochowski, Halina Wójtowicz-Młochowska

**Affiliations:** Faculty of Chemistry, Wroclaw University of Technology, Wybrzeże Wyspiańskiego 27, Wroclaw 50-370, Poland; E-Mail: halina.wojtowicz@pwr.wroc.pl

**Keywords:** oxidation, catalysts, hydrogen peroxide, *t-*butyl hydroperoxide, selenium(IV) oxide, diselenides, seleninic acids, peroxyseleninic acid, benzisoselenazol-3(2*H*)-ones, ebselen

## Abstract

A variety of selenium compounds were proven to be useful reagents and catalysts for organic synthesis over the past several decades. The most interesting aspect, which emerged in recent years, concerns application of hydroperoxide/selenium(IV) oxide and hydroperoxide/organoselenium catalyst systems, as “green reagents” for the oxidation of different organic functional groups. The topic of oxidations catalyzed by organoselenium derivatives has rapidly expanded in the last fifteen years This paper is devoted to the synthetic applications of the oxidation reactions mediated by selenium compounds such as selenium(IV) oxide, areneseleninic acids, their anhydrides, selenides, diselenides, benzisoselenazol-3(2*H*)-ones and other less often used other organoselenium compounds. All these compounds have been successfully applied for various oxidations useful in practical organic syntheses such as epoxidation, 1,2-dihydroxylation, and α-oxyfunctionalization of alkenes, as well as for ring contraction of cycloalkanones, conversion of halomethyl, hydroxymethyl or active methylene groups into formyl groups, oxidation of carbonyl compounds into carboxylic acids and/or lactones, sulfides into sulfoxides, and secondary amines into nitrones and regeneration of parent carbonyl compounds from their azomethine derivatives. Other reactions such as dehydrogenation and aromatization, active carbon-carbon bond cleavage, oxidative amidation, bromolactonization and oxidation of bromide for subsequent reactions with alkenes are also successfully mediated by selenium (IV) oxide or organoselenium compounds. The oxidation mechanisms of ionic or free radical character depending on the substrate and oxidant are discussed. Coverage of the literature up to early 2015 is provided. Links have been made to reviews that summarize earlier literature and to the methods of preparation of organoselenium reagents and catalysts.

## 1. Introduction

**Scheme 1:**
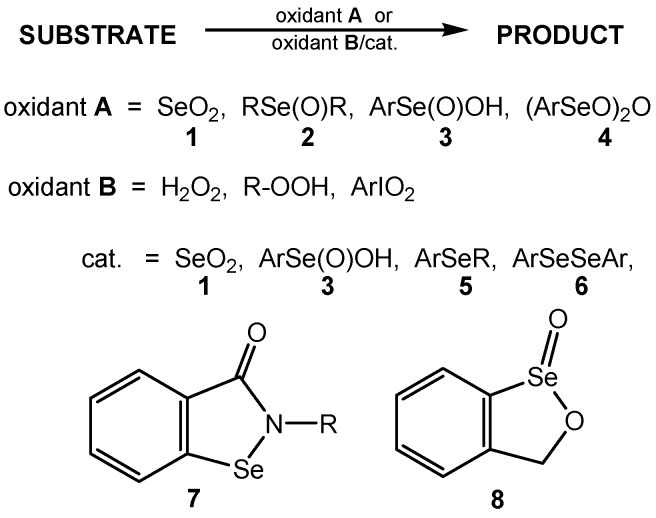
Selenium compounds as oxygen donors and oxygen-transfer agents.

Owing to the synthetic utility of the oxofunctionalization of a broad spectrum of organic substrates, oxidation is one of the fundamental processes, very often applied in contemporary organic synthesis in both research and industry. Among various oxidants selenium compounds, mainly selenium(IV) oxide (commonly named selenium dioxide, (**1**) and organoselenium compounds, presented in Scheme 1, such as selenoxides (**2**), areneseleninic acids (**3**) and their anhydrides (**4**), selenides (**5**), diaryl diselenides (**6**), cyclic selenenamides (**7**) and cyclic seleninate ester (**8**) play an important role. In earlier works selenium compounds **1**–**4** were used for oxofunctionalization of different organic substrates, mainly in stoichiometric amounts (oxidants **A**). More recently, some selenium compounds such as **1**, **3**, **5**–**8** were used in catalytic amounts, while the primary oxidants (oxidants **B**) were 30% hydrogen peroxide, *tert*-butyl hydroperoxide (TBHP), iodoxybenzene (PhIO_2_) and occasionally other oxygen donors [1,2,3,4,5,6,7,8,9,10]. Commercially available and relatively cheap peroxides of low molecular weight, such as H_2_O_2_ and TBHP, contain a high proportion of active oxygen and are environmentally friendly, because their reduction products are water or *tert*-butanol, easy to remove from reaction products and to regenerate. Since both of them are only moderately active toward most organic substrates, various promoters are used, among them, selenium compounds transferring oxygen atoms from primary oxygen donor to oxidized substrate [11,12,13,14,15,16,17,18]. The oxidation of organoselenium (also organotellurium and sulfur) compounds to diselenides, selenones, selenoxides, seleninic acids and other derivatives are illustrated in several books [9,19].

It should be noted that selenium compounds are generally regarded as toxic. It is important to realize that low volatility selenium compounds such as selenium(IV) oxide, selenoxides, diaryl diselenides, areneseleninic acids, their anhydrides, selenenamides and related compounds are odorless, but may be moderately toxic when they are absorbed. Selenium(IV) oxide forms selenous acid, a severe skin irritant, upon contact with water, sweat, or tears. The knowledge about the toxicity of broad spectrum of organoselenium compounds is still incipient despite several works on their *in vivo* toxicity [20,21,22]. For example, diphenyl diselenide (**6**, R = Ph), and 2-phenylbenzisoselenazol-3(2*H*)-one, named ebselen (**7**, R = Ph) are regarded as nontoxic. The acutely lethal dose (LD_50_) for ebselen in rats treated intraperitoneally is 400 μmol·kg^−1^ and for diphenyl diselenide 1200 μmol·kg^−1^ [23]. Some aspects that will encourage the reader to discover an unexpected green side to selenium and the chemistry connected with its organic derivatives were elucidated in review [24]. Organoselenium compounds represent a new class of reagents and catalysts in modern chemistry, green chemistry, and biological response modifiers [25,26].

Following the discovery of a broad spectrum of selenium compounds of practical importance as reagents, catalysts and intermediates, the important role they play in synthetic organic chemistry as oxygen donors and oxygen-transfer agents, judging from the numerous articles that have appeared over the last fourteen years, will be presented in this review, which covers the scientific literature in general from 2000 to the present, but includes a few significant earlier references necessary for discussion.

## 2. Selenium(IV) Oxide and Selenic(IV) Acid as Oxidizing Agents and Oxidation Catalysts

The first publication on the use of selenium(IV) oxide in oxidation reactions appeared in 1932 [27] and since then it has been applied as a versatile reagent for the synthesis of various types of organic compounds [12,23,24,25,28,29,30]. Due to its toxicity when taken orally, intense local irritation of skin and eyes, and the sometimes malodorous volatile selenium-containing by-products are formed, SeO_2_ is used in modern synthesis only when it competes favourably with other methods, provides unique reactivity or when it is used in catalytic amounts [1,2,4,12,24,25,26,27,29,30,31,32]. The TBHP/SeO_2_ or H_2_O_2_/SeO_2_ systems are more convenient to use than SeO_2_ alone, particularly when it is used in catalytic amounts, very often in 5 mol %. Reaction conditions are much milder and as a result, yields are higher with less oxidation, dehydration and rearrangement by-products. Moreover the problem of the removal of colloidal selenium is circumvented.

### 2.1. Allylic Hydroxylation

Selenium(IV) oxide-mediated oxidation of substituted olefins (Riley oxidation) is regarded as one of the most reliable and predictable methods for introducing a hydroxy group into the allylic position. The reaction reveals a very useful regio- and stereoselectivity when applied to trisubstituted olefins, producing predominantly (*E*)-allylic alcohols.

**Scheme 2:**
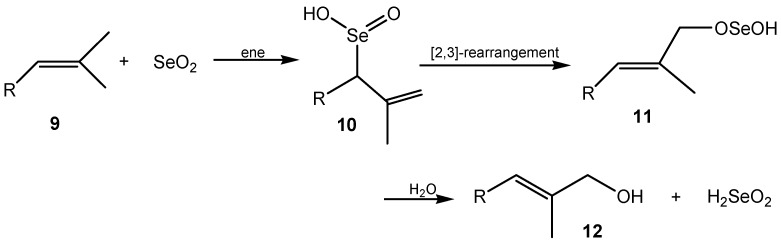
Mechanism of selenium(IV) oxide α-hydroxyalkylation of alkenes.

Selenium(IV) oxide mediates the unique allylic oxidation of alkenes **9** with usual retention of the double bond position. The mechanism of this reaction remained unclear until Sharpless and Lauer in 1972 [33] explained the selective oxidation as the result of a two-step process: an ene reaction followed by sigmatropic [2,3]-rearrangement of intermediate selenic(IV) acid **10** that give selenic(II) acid ester **11**, while the double bond returns to its original location. In the last step the ester is hydrolyzed into the allylic alcohol **12** (Scheme 2). It was postulated that in the presence of a hydroxylated solvent, e.g., water, alcohol or a carboxylic acid, the active oxidant could be selenic(IV) acid or its alkyl ester.

A comparison of the observed ^13^C and ^2^H kinetic isotope effects with the predicted values shows that the observed effects are consistent with an initial concerted ene reaction step mediated by SeO_2_. However, this comparison does not rule out the involvement of a selenic(IV) ester in the ene reaction or a stepwise reaction involving reversible electrophilic addition of HSeO_2_^+^ followed by a rate-limiting proton abstraction. B3LYP calculations strongly favour SeO_2_ over a selenic(IV) ester as the active oxidant, with a predicted barrier of 21–24 kcal·mol^−1^ lower for the reaction of 2-methyl-3-butene with SeO_2_ than that for the reaction with H_2_SeO_3_. The possibility of a selenic(IV) ester as the active oxidant is also disfavoured by the observation of oxidations in non-hydrolytic solvents. A concerted ene reaction with SeO_2_ as the active oxidant thus appears to be the major mechanistic pathway in these reactions [34,35,36].

Selenium(IV) oxide allylic hydroxylations are highly regiospecific and occur at the α-position to the more substituted carbon of the double bond with a reactivity order CH_2_ > CH_3_ > CH. When the double bond is inside a ring, oxidation occurs in the ring when possible, and in the α-position to the more substituted end of the double bond. Another synthetically very useful aspect in this conversion of the nonactivated C=C double bond into the allylic alcohol intermediate lies in its high stereoselectivity, as demonstrated in the oxidation of 1-*tert*-butyl-4-alkylidenecyclohexanes [36].

**Scheme 3:**
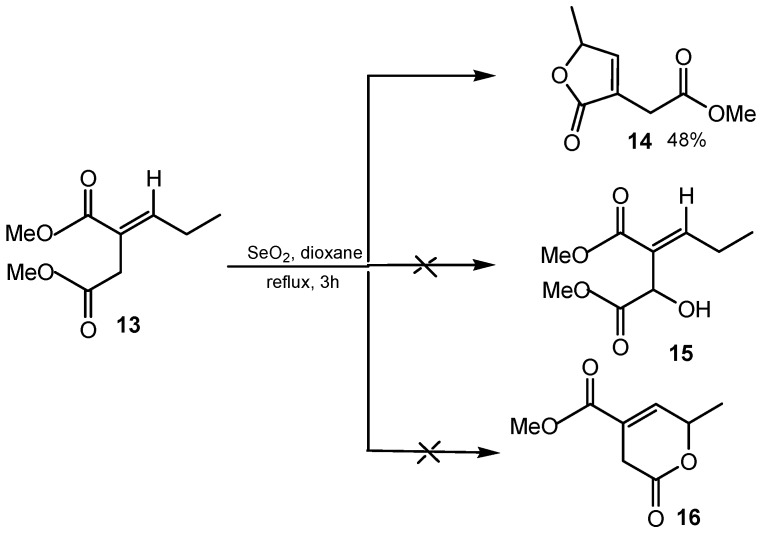
Regio- and stereoselective SeO_2_ oxidation of (*E*)-dimethyl 2-propylidenesuccinate **13**.

The (*Z*)-selective allylic alcohol formation of dialkyl alkylidenesuccinates induced by SeO_2_ has been demonstrated to accomplish one-step syntheses of several essential and fused butenolides via an unusual *E*- to *Z*- carbon-carbon double bond isomerization followed by lactonization pathway. The observed regio- and stereoselective SeO_2_ allylic oxidation protocol has also been extended to the diastereoselective total synthesis of the bioactive natural product isomint lactone, its direct conversion to mint lactone and an example of the base-catalyzed intramolecular rearrangement of γ-lactone to δ-lactone. As depicted in Scheme 3, the initial expectation was that the regioselective SeO_2_ allylic oxidation of (*E*)-dimethyl 2-propylidenesuccinate **13** would provide (*E*)-dimethyl 2-propylidene-3-hydroxysuccinate **15** or pyran skeleton **16**. The allylic oxidation of compound **13**, in the presence of a catalytic amount of SeO_2_ and *tert*-butyl hydroperoxide in *tert*-butyl alcohol/1,4-dioxane at room temperature, was not successful and the starting material remained unreacted. When the compound **13** was treated with SeO_2_ (1.60 equiv) in refluxing 1,4-dioxane, the allylic oxidation reaction was completely regioselective and provided the butenolide product **14**. This suggested that in the SeO_2_-induced transformation of dimethyl (*E*)-2-propylidenesuccinate **13** to product **14**, apart from allylic hydroxylation, the course of reaction involves a *E*- to *Z*- carbon–carbon double bond isomerization and an *in situ* intramolecular cyclization step [37].

**Scheme 4:**
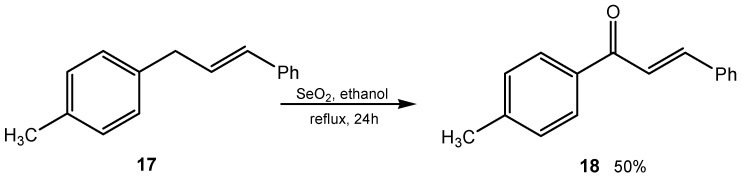
Oxidation of diarylpropene to *p*′-methylchalcone.

Selenium dioxide was found to be a reliable reagent for the direct regioselective insertion of oxygen at the allylic carbon via α-hydroxylation. Various 1,3-diarylpropenes were oxidized with SeO_2_ in ethanol in 50%–58% yield. For example, the reaction of diarylpropene **17** with SeO_2_ in ethanol gave *p*′-methylchalcone **18** in 50% yield (Scheme 4) [38].

Selenium(IV) dioxide is still used for allylic hydroxylation in several multistep syntheses and transformations of natural products, their precursors and analogues such as 6-hydrocorticosteroids, 6-β-hydroxy derivatives of progesterone and testosterone, glycospirostanes, the optically pure cyclohexenone core scyphostatin and hydroxytaxadienes [39,40,41,42,43,44,45]. Allylic oxidation of phlomisoic acid and its methyl ester by selenium(IV) dioxide occurred stereoselectively to form α-hydroxy derivatives of labdanoids [46].

Like selenium(IV) oxide alone, the reagent TBHP/SeO_2_ oxidizes alkenes, cycloalkenes and alkynes in the allylic position. Hydroxylation of cycloalkenes carrying alkyl substituents at the allylic position, takes place preferentially on the ring α-carbon atom. Oxidation of terminal alkenes results in C=C bond migration and primary allyl alcohols formation. Terminal and non-terminal vinyl fluorides have been hydroxylated regioselectively in the allylic and propargylic position adjacent to the fluorine-bearing carbon [47].

**Scheme 5:**
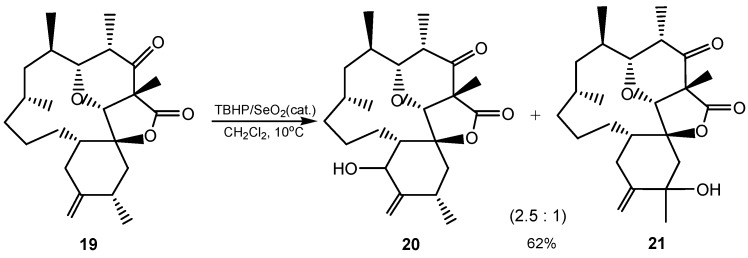
Allylic TBHP/SeO_2_ oxidation in the total synthesis of (−)-okilactomycin.

TBHP/SeO_2_ was used in the allylic hydroxylation of isolated double bonds in straight-chain hydrocarbons, e.g., monounsaturated fatty acids, esters and alcohols. Either allylic position was hydroxylated or both positions reacted, to give dihydroxy isomers. Yields of monohydroxy compounds in which the OH group was between the double bond and C(1), were usually higher than those in which the OH group was between the double bond and the methyl terminus. When an α-methylene group is oxidized, the reaction proceeds under mild reaction conditions [1,2,9,17,43,48]. For example, TBHP/SeO_2_ oxidation of compound **19** in multistep synthesis of (−)-okilactomycin gave both possible isomers **20** and **21** (Scheme 5) [49]. A mixture of taxadienes (87% of taxa-4(5),11(12)-diene and 13% of taxa-4(20),11(12)-diene), was subjected to oxidation with TBHP/SeO_2_ and stoichiometric amounts of selenium(IV) oxide. In both cases, the expected α-hydroxylation products were isolated [50].

Urea-hydrogen peroxide (UHP), in the presence of catalytic quantities of SeO_2_, has successfully led to the allylic oxidation of alkenes while keeping the other chemical functionalities intact. The reaction conditions are environmentally benign as both UHP and microwave irradiation are considered eco-friendly green chemistry routes [51]. Sesquiterpene lactones like dehydrocostus lactone and isoalantolactone were subjected to allylic hydroxylations with SeO_2_ in combination with urea hydrogen peroxide in polyethylene glycol to form allylic alcohols. The reactions were more selective, and the yields were higher than reactions with TBHP/SeO_2_ in dichloromethane [52,53]. Good results were obtained where TBHP/SeO_2_ was used as the oxidant for methyl geraniate, whereas farnesyl acetate, a terpene possessing three different double bonds, yielded only 24% of the desired alcohol [54].

The TBHP/SeO_2_ oxidation of some simple cycloalkenes produced, in addition to the expected allylic alcohols, allylic *t*-butyl ethers and *t*-butyl peroxides. For cyclohexene, the major products were the ether and peroxide. As the ring size increased, the yields of alcohols increased and those of ethers and peroxides decreased. When the oxidation was carried out in the presence of hydroquinone, the peroxides were not observed, although the yields of alcohols and ethers remained unaffected. Consequently, a free-radical pathway has been proposed. Another mechanism, involving a carbocation intermediate, can be also envisaged to explain the isolation of isomeric allylic esters, resulting from TBHP/SeO_2_ oxidation of pinene derivatives [55]. Selenium(IV) oxide associated with *N*-methylmorpholine *N*-oxide was successfully applied as very efficient hydroxylating agent for monocyclic unsaturated terpenoids. An advantage of this feature is high conversion of the substrate (67%–100%) and stereospecific functionalization [56].

In contrast with alkenes, alkynes show a strong tendency to α,α′-dihydroxylation upon reaction with TBHP/SeO_2_. The oxidation of different acetylenes allowed assignment of the reactivity sequence CH_2_=CH > CH_3_. Alkynes bearing one methylene and one methine substituent afforded the enynone as the major product [57]. Despite the fact that stereochemical aspects and mechanism of the reaction are known, so far it has not shown any synthetic utility.

### 2.2. 1,2-Dihydroxylation of Alkenes

**Scheme 6:**
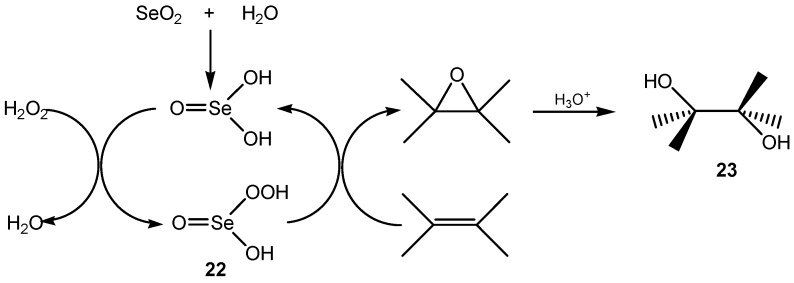
Hydrogen peroxide *trans*-1,2-dihydroxylation of alkenes catalyzed by SeO_2_.

A number of alkenes were *trans*-dihydroxylated with 30% aqueous hydrogen peroxide in the presence of 20 mol % of SeO_2_ at room temperature. The isolated yields of the diols were in the 55%–88% range. Cyclic, acyclic, terminal and internal alkenes were smoothly converted to their corresponding vicinal diols and no mono α-hydroxylation or α-oxygenation to aldehydes or ketones was observed. It was found, that aliphatic alkenes exhibited better results than their aromatic analogues and sterically hindered double bonds exhibited poorer yields compared with less hindered ones. When arylidenemalononitriles were used as substrates, they produced the corresponding carbonyls due to the presence of the two electron-withdrawing groups on one terminal of the olefins. The peroxyselenic(IV) acid **22**, formed *in situ* from hydrogen peroxide and selenic(IV) acid, is responsible for the epoxidation of alkenes, which in the presence of water and selenic(IV) acid forms the corresponding *trans*-1,2- diols **23** (Scheme 6) [58].

A synthetic method for some arylpyridines involved H_2_O_2_/SeO_2_ dihydroxylation and methoxyhydroxylation of 4-aryl-1,2,3,6-tetrahydropyridines. This facile strategy was also used to synthesize several hydroxylated 4-arylpyridines, 3-hydroxy-4-arylpyridines, and 3,4-diarylpyridines [59]. A novel selenium(IV) oxide-mediated dihydroperoxidation of 3-aryl-1,4,5,6-tetrahydropyridine was also examined [60].

Long chain alkenes and unsaturated acid esters oxidized with H_2_O_2_/SeO_2_ at ambient temperature gave, depending on the reaction time, vicinal diols, selenite esters and epoxides. For methyl oleate, after a short reaction time (4 h) the epoxide was produced, while the time was prolonged for 24 h, ester accompanied with diol was a major product. It supported the hypothesis that the product sequence is epoxides → selenite esters → vicinal diols [61].

### 2.3. Oxidation of the Methyl and Methylene Groups

Selenium(IV) oxidation of methylpyridines, methylquinolines, methylphenanthrolines, methylpterines and other heterocycles by heating with selenium(IV) oxide in 1,4-dioxane, is a good way to synthesize the corresponding aldehydes. The 2-methyl group is more susceptible toward oxidation than a 4-methyl group, e.g., in 2,4-dimethylquinoline or its oxide, the 2-methyl group was oxidized preferentially [1,2,9].

Conversion of methyl and methylene groups to formyl and keto groups by oxidation with stoichiometric amounts of selenium(IV) oxide still remains an attractive route in comparison with other methods. Some examples show the use of this reagent in the synthesis of aldehydes and ketones. The 7-methyl group of 2-acetylamino-7-methyl-1,8-naphthyridine was oxidized with SeO_2_ in 1,4-dioxane to the corresponding formyl group in 75% yield [62]. Oxidation of 4-amino-2-methyl-5,10-dioxo-1,5,10,10a-tetrahydrobenzo[g]quinoline-3-carbonitrile with selenium(IV) oxide provided 4-amino-2-formyl-5,10-dioxo-1,5,10,10a-tetrahydrobenzo[*g*]quinoline-3-carbonitrile in 76% yield [63]. Refluxing dioxane solutions of 6-methyl-2,4-dioxypyrimidine with selenium(IV) dioxide or with selenic(IV) acid in acetic acid, was found to afford orotic aldehyde in 50% or 62% yield, respectively [64]. A series of other important heterocyclic aldehydes and ketones were synthesized by microwave- assisted selenium(IV) oxide oxidation [65,66]. The crucial step in synthesis of the antibiotic caerulomycin E was selenium(IV) oxide oxidation of a methyl group to a formyl group in 4-methoxy-6-methyl-2,2′-bipyridine [67]. On the way to synthesize verdamycin C2, the corresponding aldehydes were obtained by oxidation of allylic primary azides of 2-substituted dihydro[2*H*]pyrans with SeO_2_ [68].

The relative ease of overoxidation to carboxylic acid permits ones to convert methyl groups into carboxylic acids directly [69]. In some cases it is the most serious disadvantage of the reagent. The oxidation can be stopped at the first stage in the presence of acetic anhydride. The intermediate selenic(II) ester is re-esterified and the acetate derived from the primary alcohol is formed [1,2,9]. The TBHP/SeO_2_ reagent allowed the oxidation of activated methyl groups of *N*-heterocyclic compounds under milder conditions than SeO_2_ alone, without the formation of the over-oxidized carboxylic acids [70]. A subsequent oxidization of the formyl group to carboxylic group, which underwent spontaneous decarboxylation, was applied for selective elimination of the methyl substituent from azaheterocyclic compounds, e.g., 7-methylxantopterin [71]. Mono- and diformyl-4*H*-pyranones were obtained in suitable yields using SeO_2_ as a stoichiometric methyl group oxidant. In this process selenium(IV) dioxide was reduced to elemental selenium which was reclaimed by reaction with nitric acid, and the selenic(IV) acid formed was used for oxidation in the next oxidation cycle [72].

**Scheme 7:**
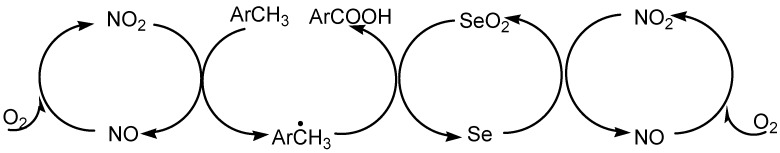
The mechanism of nitrogen oxides/SeO_2_ benzylic oxidations.

Oxidation of toluenes to benzaldehydes was carried out by the formation of the active oxidant obtained by treatment of SeO_2_ with TBHP, prior to addition to the substrate. However, the oxidation of toluenes to benzaldehydes, in the presence of other oxidizable groups, is most often troublesome. The oxidation of benzylic groups to the corresponding carboxylic acid functionality is mediated by a combination of selenium(IV) oxide (or elemental selenium) and nitrogen oxides, while the stoichiometric oxidant is dioxygen. 2-Methylnaphthalene reacted completely after 4 h at 160 °C, forming 2-naphthalenecarboxylic acid in 80% yield. Under the same reaction conditions 4-pyridine-carboxylic acid was obtained from 4-methylpyridine in 94% yield. The proposed mechanism is summarized in Scheme 7. Nitric oxide (NO) is oxidized rapidly and spontaneously to nitrogen dioxide (NO_2_). The latter has two functions. First, it abstracts one of the benzylic hydrogens from the substrate to form the benzyl radical, nitric oxide and water. Second, it oxidizes selenium to SeO_2_ (or H_2_SeO_3_) formed in the presence of water), which then selectively oxidizes the benzyl radical to the corresponding aldehyde and finally to the acid [73].

**Scheme 8:**
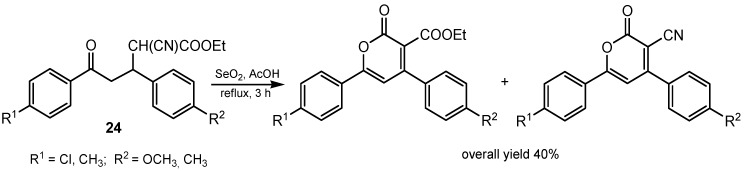
Selenium(IV) oxide oxidation of ethyl 3,5-diaryl-2-cyano-5-oxapentanoate.

Oxidation of the methylene group of enolizable ketones with SeO_2_ in acetic acid or in 1,4-dioxane provides α-oxidation to α-diketones. Indolones oxidized in this way gave α-diketones in 51%–95% yields [74]. The chemoselective reactions of selenium(IV) oxide with differently substituted 1,4-adducts derived from substituted arylidene acetophenones **24** were also described. This reaction has been shown to be dependent on the nature of the substituent present, leading to different products by α-oxidation to diketones, α-oxidation followed by dehydrogenation or followed by dehydrogenation, enolization, and cyclization (Scheme 8) [75].

**Scheme 9:**
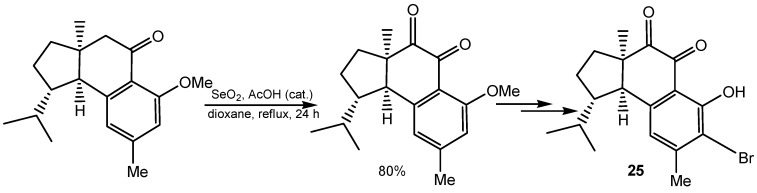
Synthesis of 6-*epi*-(−)-hamigeran B.

In one of the steps of the total synthesis of novel natural product 6-*epi*-(−)-hamigeran B (**25**), isolated from a poecillosclerid sponge, α-methylene group in cyclohexanone moiety was oxidized to an α-keto group in 80% yield with SeO_2_ in dioxane-water in the presence of catalytic amounts of acetic acid (Scheme 9) [76].

**Scheme 10:**
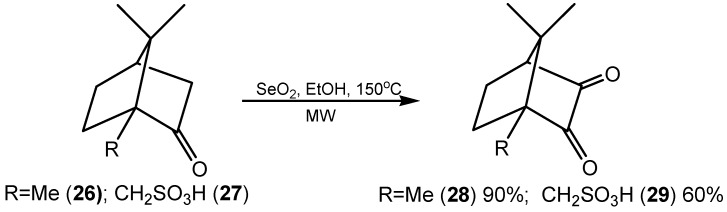
SeO_2_ oxidation of camphor derivatives.

An improved procedure for the microwave-assisted selenium(IV) oxide oxidation of aryl methyl ketones to aryl glyoxals and diarylethanones to 1,2-etanediones, was elaborated using dimethylsulfoxide as solvent [77,78]. Under focused monomode microwave irradiation, camphor (**26**) and camphor sulfonic acid (**27**) (Scheme 10), and also camphor sulfonylimine, were oxidized with SeO_2_ to the respective 3-oxocompounds **28** and **29**, with the further advantage of an almost quantitative precipitation of elemental selenium which was easy to remove from the reaction mixture by filtration [79].

For synthesis of the 1,2,3-trione compounds from 1,3-diketones H_2_O_2_/SeO_2_ was used as oxidative agents and THF-H_2_O as solvent. This method provides better yields in comparison with other similar methods [80].

### 2.4. Dehydrogenation and Oxidative Bond Cleavage

**Scheme 11:**
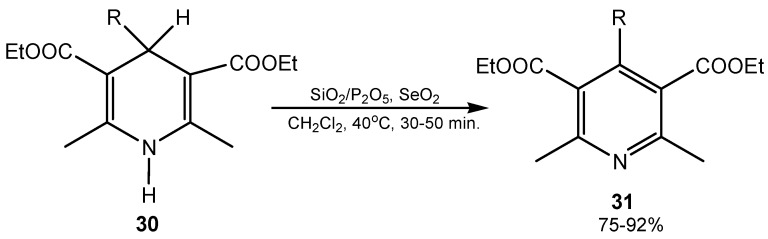
Selenium(IV) oxide-mediated aromatization of Hantzch 1,4-dihydropyridines.

Elimination reactions, including dehydrogenation, are favoured when a strong conjugated system can be formed, and are often applied for aromatization of unsaturated carbocyclic and heterocyclic rings. For this purpose selenium(IV) oxide is a good reagent. Some examples have been given in reviews [4,9]. More recently 1,4-dihydropyridines were aromatized in 87%–98% yields using stoichiometric SeO_2_ at ambient temperature [81]. Aromatization of Hantzch 1,4-dihydropyridines **30** to the corresponding pyridines **31** was carried out in high yield under heterogeneous conditions using silica-supported P_2_O_5_ and SeO_2_ as the stoichiometric oxidant (Scheme 11) [82]. In one of the steps of the synthesis of antimicrobial pyrimidine-5-carboxylates, the 3,4-dihydropyrimidine ring was aromatized to a pyrimidine ring using SeO_2_ [83]. Microwave-assisted dehydrogenation of 4,6-diaryl-4,5-dihydropyridazin-3(2*H*)-ones to 4,6-diarylpyridazin-3(2*H*)-ones, with SeO_2_ in the solid state is a good way for the aromatization of the heterocyclic ring because of the short reaction times and high yields of products (79%–84%) [84].

A new method for the synthesis of 5-azaindole involves [3+2] dipolar cycloaddition between nitriles and a 3,4-cyclopropanopiperidine, followed by SeO_2_ oxidation [85]. It affords the target compounds in moderate to excellent yields. Selenium(IV) oxide oxidation of cholesterol reveals a solvent-dependent product selectivity and provides a facile one-pot synthesis of its derivatives, including aromatic analogs of naturally occurring ergosterol [86].

Attack of selenium(IV) oxide at activated positions can lead to oxidative bond cleavage when appropriate leaving groups are present. Aryl propargyl ethers undergo oxidation at the α-alkynyl position to afford a phenolic species and propargyl aldehyde. The analogous aryl allyl ether fragmentations occur in somewhat lower yields. (Hydroxyaryl)pyrazolines were oxidized with nitrogen extrusion to afford 2-hydroxychalcone [9,30].

It was found that during the H_2_O_2_/SeO_2_ oxidation of lupanone oxime, nitrogen atom elimination takes place to yield two different lactones. Contrary to the earlier observation that the nitrogen of the oximes remains intact during its reaction with only SeO_2_, in this case the addition of H_2_O_2_ has been found to remove the nitrogen during the course of the reaction [87]. Selenium(IV) oxide-catalyzed oxidation of acetic acid hydrazide by bromate gives acetic acid as the oxidation product. Kinetic studies have shown that the reaction proceeds through formation of a complex between the catalyst and hydrazine which will be oxidized by the oxidant in a rate determining step [88]. Selenium(IV) oxide also catalyzes the oxidation of nicotinic acid hydrazide (NIH) by bromate in hydrochloric acid medium. The NH_2_ group of the hydrazoic moiety and pyridine nitrogen of the NIH forms protonated species which are involved in two ion pair complexes with the oxidant in a prior equilibrium. In the case of the uncatalyzed reaction the complex with the protonated hydrazoic moiety decomposes to give the corresponding acyl diimide intermediate, while that of the pyridine nitrogen decreases the rate of reaction. In the presence of selenium(IV) oxide as catalyst, the NIH reduces the catalyst to H_2_SeO_2_, which is oxidized by the oxidant to complete its catalytic cycle. The reaction product is found to be nicotinic acid and there is no intervention of any free radicals [89].

### 2.5. Oxidative Cyclization and Ring Transformations

Selenium(IV) oxide reacts with semicarbazones of aldehydes or ketones under heating in acetic acid or dioxane. An oxidative ring closure takes place and 1,2,3-selenadiazoles are produced. This method has long been used for synthesis of these heterocycles, and has a practical value because these species are utilized as useful synthetic intermediates through a variety of thermal and photochemical decomposition reactions with the loss of nitrogen and/or selenium. More recently, ethoxycarbonyl hydrazones and tosylhydrazones were also used for the reaction with SeO_2_ in acetic acid. A number of works on the synthesis and use of 1,2,3-selenadiazoles has been discussed in reviews [9,90,91,92] and in more recently published original papers [93,94,95,96,97,98,99,100]. Synthesis of novel benzopyrano-1,2,3-selenadiazole and spiro[benzopyrano]-1,3,4-thiadiazoline derivatives as possible antitumor agents is an example [96].

Most ring syntheses of 1,2,5-selenadiazoles and their fused systems such as 2,1,3-benzoselenadiazoles involve the reaction of 1,2-diamines with selenium(IV) oxide or selenium oxychloride [9,90,93,101]. For example, 1,2-phenylenediamine and different ring-substituted derivatives were cyclized to 2,1,3-benzoselenadiazoles in good to excellent yields. Some of them are valuable synthetic intermediates [102,103].

**Scheme 12:**
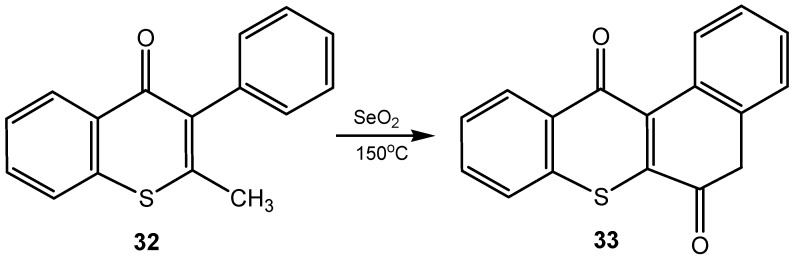
Intramolecular Friedel-Crafts reaction-oxidation of indeno[2,1-*b*]tiochromene-6,11-dione promoted by selenium(IV) oxide.

Various substituted 4-hydroxyimidazoles were obtained in a single-pot synthesis by SeO_2_ oxidation of 1,3-diazabuta-1,3-dienes [104]. Selenium(IV) oxide-mediated oxidative amidation of arylglyoxals with secondary amines, carried out under microwave irradiation gave the α-keto amides, followed by an acid-promoted deprotection and cyclization to afford quinoxalinones and diazepinones in moderate to good yields. For example, in the presence of SeO_2_ in CH_2_Cl_2_, reaction of PhCOCHO and 2-(BocNH)C_6_H_4_NHBu-*i*, followed by treatment with trifluoroacetic acid gave quinoxalinone in 65% yield [105]. A simple method to preparation of indeno[2,1-*b*]thiochromene-6,11-dione (**33**) from 2-methyl-3-phenylthiochromen-4-one (**32**) involves an intramolecular Friedel-Crafts reaction-oxidation cascade. It starts from SeO_2_ oxidation of 2-methyl group into formyl group which subsequently acylate of adjacent benzene ring, to give a new cyclopentanone ring (Scheme 12) [106].

**Scheme 13:**
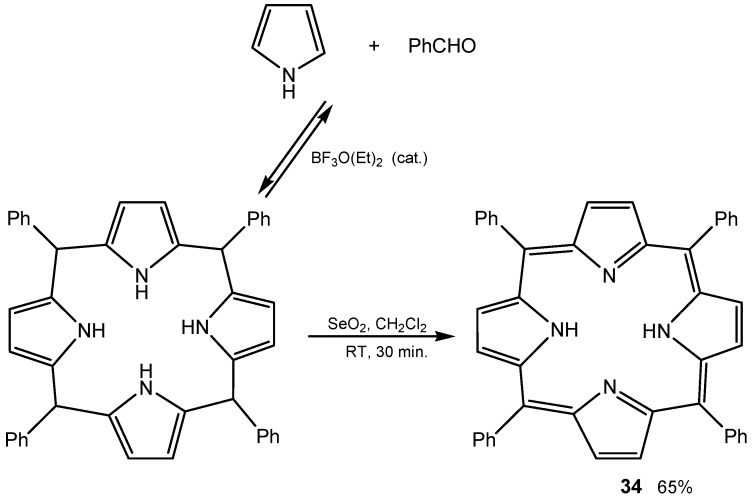
Synthesis of tetraphenylporphyrin through condensation of pyrrole and benzaldehyde followed by oxidation with SeO_2_.

The oxidative step of the one-flask synthesis of *meso*-tetraphenylporphyrin (**34**, Scheme 13), and other tetraarylporphyrins, was conducted with heterogeneous SeO_2_ as oxidant instead of the usual quinones DDQ or *p*-chloranil. The simplicity of the workup, allied with mild reaction conditions, makes this method a good option for the synthesis of this kind of compounds [107].

Selenium(IV) oxide affects the oxidative ring contraction of some six-membered sulfa- and selenaheterocyclic rings (e.g., in selanachromene or tiochromene), to a five-membered ring [9]. The reaction of 2,3-dihydro-1*H*-1,2-diazepino[3,4-*b*]quinoxaline with SeO_2_ in AcOH/H_2_O resulted in ring transformation to give the 1,4-dihydro-4-oxopyridazino[3,4-*b*]quinoxaline [108], while the result of oxidation of dialkyl-3*H*-azepines depends on the position of alkyl groups and different compounds, among them ring-opening and ring-contraction products are formed [109].

### 2.6. Miscellaneous Oxidative Transformations

**Scheme 14:**
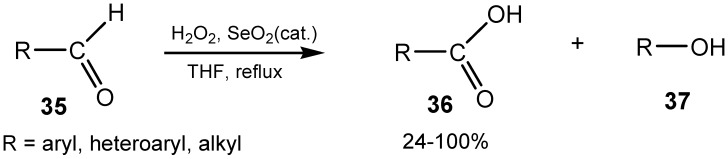
Selenium(IV) oxide catalyzed oxidation of aldehydes.

There are several reports on the use of SeO_2_ as a stoichiometric oxidant, and more often as a catalyst, for oxidative transformations of different organic compounds, other than those mentioned in the previous sections. Aromatic aldehydes and ketones treated with selenium(IV) oxide react in different ways. Hydrogen peroxide with a catalytic amount of SeO_2_ promotes the Bayer-Villiger reaction of various aromatic aldehydes possessing hydroxy or methoxy substituents. Oxidation of aromatic aldehydes **35** having no substituents or these ones bearing methyl groups lead to the corresponding carboxylic acids **36**. Similar results were found for aromatic aldehydes having one or two electron-withdrawing groups, although for disubstituted ones the reaction proceeded more slowly. In all these cases, arenecarboxylic acids were isolated in yields of above 83%. Even benzaldehydes having the electron-donating methoxy group in the *ortho* or *o-* or *p-*position produced substantial amounts (44%–46%) of acids **36** beside phenols **37**. Oxidation of aromatic dialdehydes resulted in production of dicarboxylic acids in 80%–93% yields. Aliphatic aldehydes undergo oxidation to carboxylic acids substantially faster than aromatic ones and carboxylic acids were produced in 80%–100% yield (Scheme 14) [110]. Treatment of acetophenone and acetone with hydrogen peroxide in tertiary butanol with selenium dioxide as catalyst gave phenylacetic acid and propionic acid with selectivities of 80% and 97%, respectively [111].

**Scheme 15:**
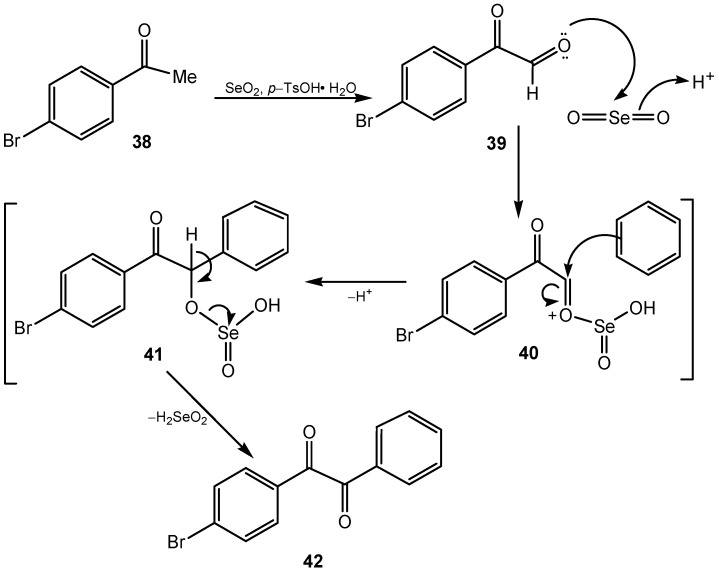
Proposed mechanism for the one-pot synthesis of unsymmetrical benzils.

Selenium(IV) oxide promotes C-C bond formation. A direct and efficient protocol for the preparation of unsymmetrical and heteroaryl 1,2-diketones through oxidative coupling between the α-carbon atom of the aromatic ketone with unactivated arenes in the presence of SeO_2_ and *p*-TsOH·H_2_O was reported. In this way unsymmetric benzils were obtained in good yield (38%–75%). A plausible mechanism is shown in Scheme 15. The oxidation of acetophenone **38** to glyoxal **39** by SeO_2_, is followed by the preferential formation of an O–Se bond through the carbonyl oxygen atom of the aldehyde group in the presence of *p*-TsOH·H_2_O, to give the intermediate **40**. The activating effect of the keto group and the formation of the O–Se bond generate a strong electrophilic centre at the aldehyde carbon atom of **40**, which is highly susceptible to attack from electron-rich arenes to give the selenite intermediate **41**. Oxidative decomposition of **41** led to the final product **42** [112].

**Scheme 16:**
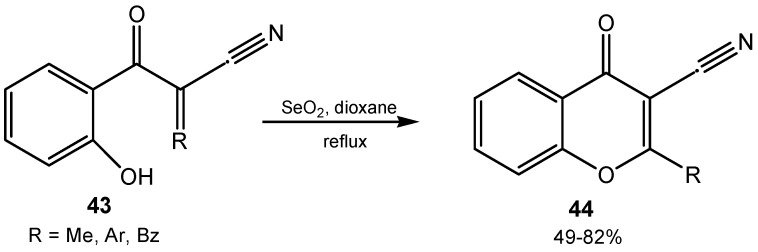
Synthesis of 2-substituted 2-cyanochromones.

Oxidative cyclization of 2-hydroxybenzoylacrylonitriles **43** with selenium(IV) oxide was the final step of synthesis of 2-alkyl-, 2-aryl-, and 2-heteroaryl-3-cyanochromones **44** (Scheme 16) [113].

**Scheme 17:**
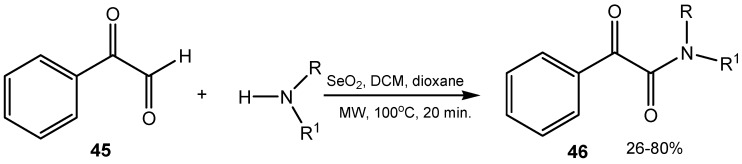
Selenium(IV) oxide-mediated synthesis of α-ketoamides.

Oxidative coupling of racemic 1-ethoxy-1-oxophosphindolin-3-one and its 5-CF_3_-derivatives with SeO_2_ furnishes 1,1′-diphosphaindigo derivatives [114]. A facile synthetic approach for the synthesis of α-ketoamides **46** by reaction of selenium(IV) oxide-mediated oxidative amidation between arylglyoxals **45** and secondary amines accelerated with microwave irradiation was described (Scheme 17) [115].

Differently ring-substituted anilines were oxidized to nitroso compounds or azoxybenzenes using hydrogen peroxide and various catalysts, including selenium(IV) oxide. As has been shown for methyl 4-aminobenzoate the result depends strongly on the solvent. Treatment of this compound with H_2_O_2_/SeO_2_ in methanol at room temperature furnishes exclusively the azoxybenzene. By conducting the oxidation with the same reagent in aprotic, non-polar dichloromethane, the nitrosoarene was a major product [31,116].

Selenium(IV) oxide alone and H_2_O_2_/SeO_2_ oxidize sulfides to sulfoxides and/or sulfones . The C–N bond in some endocyclic sulfonamides can be split off and converted into a carbonyl group by oxidation with SeO_2_ followed by hydrolysis. Symmetrical and unsymmetrical diketones are readily prepared by this method [117]. Selenium(IV) oxide catalyzed oxidation of benzotriazole thioethers by H_2_O_2_, proceeds selectively and yields sulfoxide only while oxidation by H_2_O_2_ without catalyst is not selective and sufoxides and sulfones are formed [118].

Selenium containing sub-valence heteronuclear peroxotungstate, [C_18_H_37_N(CH_3_)_3_]_4_[H_2_Se^IV^_3_W_6_O_34_] was found to be a good catalyst for hydrogen peroxide oxidation of dibenzothiophene to its corresponding sulfone under mild biphasic conditions [119]. Another selenium(IV)-containing dinuclear peroxotungstate, [(*n*-C_4_H_9_)_4_N]_2_[SeO_4_{WO(O_2_)_2_}_2_] was used for the hydrogen-bond-assisted epoxidation of homoallylic and allylic alcohols with H_2_O_2_. This system has an advantage over H_2_O_2_/SeO_2_ system such as high yields, selectivity to epoxy alcohols, efficiencies of H_2_O_2_ utilization (use of 1 equiv. H_2_O_2_, or organic hydroperoxide, with respect to a substrate instead of excess) and mild reaction conditions [120].

## 3. Organoselenium Compounds as Oxidizing Agents and Oxidation Catalysts

Until the early 1970s only selenium(IV) oxide (for instance, in allylic oxidations) and elemental selenium (as dehydrogenating agent) had been applied for synthetic purposes. Following the discovery of a broad spectrum of organoselenium compounds of practical importance as reagents, catalysts and intermediates, they began to play an important role in synthetic organic chemistry, judging from the numerous original papers, books and review articles that have appeared over the years [1,2,3,4,5,6,7,8,9,10,17]. Because only a few organoselenium compounds which can be used as stoichiometric oxidants or catalyst are commercially available and some of them are expensive, the methods for their preparation have been elaborated in detail. Moreover, most of them has been applied in catalytic amounts only and some of them can be recovered and reused.

### 3.1. Selenides and Selenoxides

Synthetic applications of selenides and selenoxides as reagents or oxygen-transfer catalysts are less common than the use of selenium(IV) oxide and are limited to only a few cases. Selenoxides and selenides have been used as catalysts in both H_2_O_2_/R^1^Se(O)R^2^ or H_2_O_2_/R^1^SeR^2^ systems, since the selenoxides are generated *in situ* from selenides and returned to the reaction cycle. It has long been known that the selenoxides, particularly diphenyl, bis(*p*-methoxyphenyl) and dimethyl, are mild reagents and catalysts for oxidation of various organic compounds such as alkenes, alcohols, thiols, sulfides, phosphines, hydrazides, amines, catechols, halomethylarenes and trivalent phosphorus compounds [4,6,9,17,121].

**Scheme 18:**
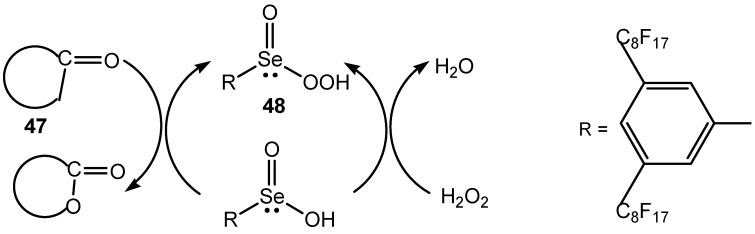
Selenium catalyzed oxidation of carbonyl compounds with aqueous hydrogen peroxide.

In recent years some new selenides and selenoxides were applied as catalysts of oxidative transformations of different organic compounds. 2-Carboxyphenyl phenyl selenide was successfully used as catalyst for hydrogen peroxide oxidation of sulfides into sulfoxides and/or sulfones [122]. Benzyl 3,5-bis(trifluoromethyl)phenyl selenoxide is an efficient catalyst for the epoxidation of various olefinic substrates and the Baeyer–Villiger oxidation of aldehydes and ketones with hydrogen peroxide [123]. Another oxygen-transfer, easy-to-regenerate, catalyst 2,4-bis(perfluorooctyl)phenyl butyl selenide was used for epoxidation of alkenes by 60% hydrogen peroxide in fluorinated solvents [124]. Oxidation of aldehydes and ketones **47** under mono-, bi- or triphasic conditions with 3,5-bis(perfluoro-octyl)phenyl butyl selenide gave carboxylic acids or carboxy esters, respectively. The active intermediate was the corresponding bis(perfluorooctyl) benzeneperoxyseleninic acid **48** (Scheme 18) [125].

**Scheme 19:**
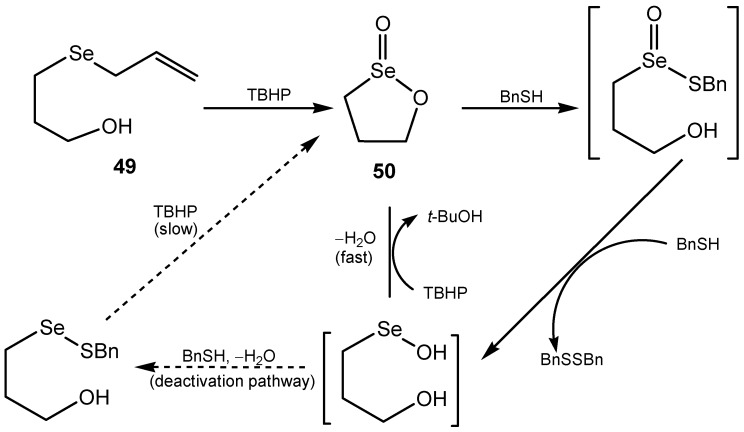
Allyl selenide **49** as a catalyst for TBHP oxidation of benzylthiol to benzyldisulfide.

It has been found that allyl selenides are good catalysts for the TBHP oxidation of benzylthiol to benzyldisulfide although there is no experience of their use in practical synthesis. Evidence has been put forth showing that 3-hydroxypropyl allyl selenide **49** acts in biomimetic way, like the enzyme glutathione peroxidase, being a precatalyst which undergoes a series of rapid oxidations and sigmatropic [2,3]-rearrangement steps to form a cyclic seleninate ester **50**. This active intermediate is involved in a catalytic cycle as shown in Scheme 19. Aromatic cyclic seleninate esters and spirodioxyselenuranes, although less active, can act in a similar way [126,127,128,129]. Kinetic study results have revealed that in the presence of H_2_O_2_ selenoxides are converted to hydroxy perhydroxy selenanes HOSe(R^1^,R^2^)OOH, which are kinetically better oxidizing agents than selenoxides [130]. The evaluated ability of PhSeZnCl to catalyze the oxidation of thiols to disulfides was also correlated to a catalytic glutathione peroxidase-like activity and in the same work, the first evidence that vinyl phenylselenides can promote the oxidation of thiols reducing hydrogen peroxide through the formation of a selenoxide intermediate was also reported [131]. Two chlorooxaselenuranes were used for oxidation of sulfides into sulfoxides [132].

**Scheme 20:**
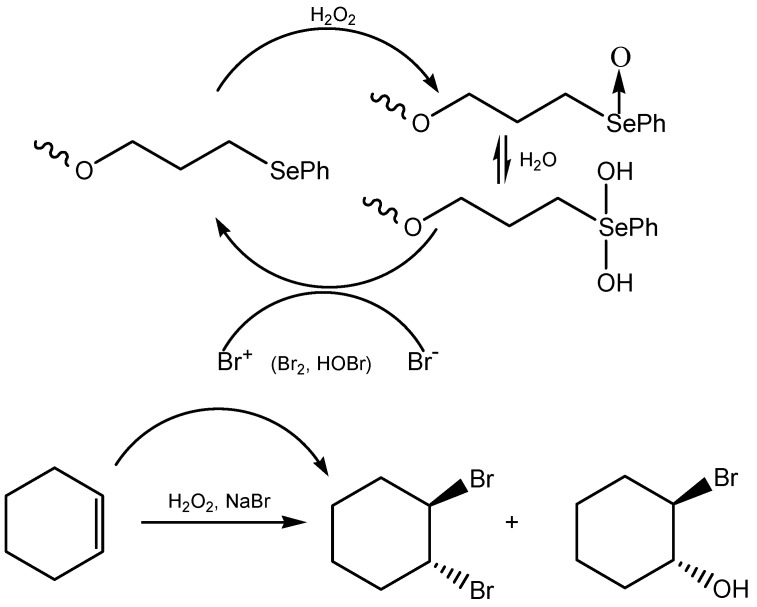
Oxidation of bromide ion with dendrimeric polyphenyl selenide.

Dendrimeric polyphenyl selenide can catalyze the oxidation of bromide with hydrogen peroxide for subsequent reaction with alkenes (Scheme 20) [133]. A dendrimer with twelve PhSe groups showed an autocatalytic effect which resulted in the turnover numbers above 6 × 10^4^. The reaction is initiated by the bromonium cation generated in the uncatalyzed background reaction [129,134]. An impressive catalyst for the bromination of arenes and for bromolactonization is (4-hydroxymethyl) phenyl selenoxide. The catalyst is easily separated from the reaction mixture by filtration and can be reused without loss of activity [135].

### 3.2. Seleninic Acids and Their Derivatives

In the 1970s and 1980s Barton, Ley and Back recognized the synthetic utility of benzeneseleninic acid C_6_H_5_SeOOH (**51**) and anhydride (C_6_H_5_Se)_2_O (**52**) as oxidants, or catalysts of hydrogen peroxide oxidation. A couple of years later, 2-nitro- and 2,4-dinitrobenzeneseleninic acids (**53** and **54**) were also successfully employed as oxidants and catalysts for hydrogen peroxide oxidation of various organic compounds. The methods for their preparation and use in synthesis have been the subject of reviews [1,4,6,8,9,17,136,137,138]. They are easily prepared by oxidation of the corresponding diselenides with ozone, TBHP or H_2_O_2_. The acid **51** and anhydride **52** are commercially available reagents. The acids **51**, **53**, **54** and anhydride **52** show some similarity to selenium(IV) oxide in their behavior, but often react more cleanly, making isolation of the products less troublesome. Moreover, the formation of evil-smelling by-products is minimized and formation of red selenium is generally avoided.

In the older works it has been revealed that oxidation of phenols with acid **51** provides an useful route to 1,4-quinones, while the use of anhydride **52** affords chiefly the corresponding 1,2-quinones. When the reaction was carried out in the presence of hexamethyldisilazane, a reactive intermediate, namely oligomeric (RSeN)_4_ was formed and then oxidized a phenol to a selenoiminoquinone. The reduction of selenoiminoquinones gave *o*-hydroxyanilines or their derivatives. Polystyrene-supported acid **51** was employed as catalyst for TBHP oxidation of benzyl and allyl alcohols into aldehydes and phenols into quinones. Alkyl groups in alkylarenes and alkylheteroarenes were oxidized with anhydride **52** into formyl or keto groups. A variety of carbonyl compounds were dehydrogenated to the corresponding α,β-unsaturated derivatives. When iodoxybenzene (PhIO_2_) or 3-iodylbenzoic acid was used a stoichiometric oxidant, anhydride **52** or its precursor, diphenyl diselenide, was employed in a catalytic amount. Anhydride **52** was also used as a reagent for α,β-dehydrogenation of lactones and lactams, but in some cases the lactams were oxidized to imides. Acid **51**, and more often anhydride **52**, were employed for oxidation of sulfides, thioketones and thioacetals, and for oxidation of nitrogen compounds such as hydrazines, hydrazides, amines, imines, hydroxylamines, and enamides [6,7,8,9].

**Scheme 21:**
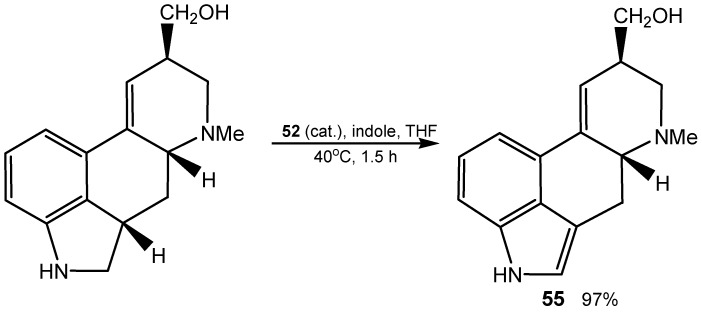
Dehydrogenation with anhydride **52** in the total synthesis of (±)-lysergol.

Oxidation of indolines affords the corresponding indoles, and this method was successfully applied to the final step in total synthesis of ergot alkaloids, among them (±)-lysergol **55** (Scheme 21) [139].

**Scheme 22:**
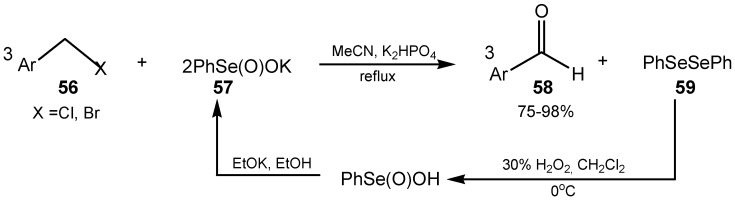
Oxidation of halomethylarenes with potassium benzeneseleninate.

Potassium benzeneseleninate **57** was employed for the oxidation of halomethylarenes **56** into aldehydes **58**. Diphenyl diselenide (**59**) resulting from this reaction can be quantitatively converted into salt **57** and reused (Scheme 22) [140].

Acids **53**, **54** and related diselenides were applied as catalysts for hydrogen peroxide and TBHP oxidation of different groups of aldehydes and aryl methyl ketones into phenol formates or acetates which are subsequently hydrolysed to phenols in one-pot procedures. In the same way the vinyl formates, accompanied by the products of their subsequent transformations, were obtained from α,β-unsaturated aldehydes. Epoxidation of styrene and its analogues with hydrogen peroxide catalyzed by acid **53** also has synthetic value. The same reagent was used for practical conversion of *N*,*N*-dimethylhydrazones into nitriles, while aldoximes in the presence of primary or secondary alcohols produced carboxy esters. Pentaflurobenzeneseleninic acid and 2-(*N*-oxide)pyridineseleninic anhydride are reagents used for the oxyfunctionalization of the allylic position in alkenes and oxidation of the hydroxymethyl group into the formyl group [6,9].

In conjunction with iodoxybenzene as reoxidant an easily accessible perfluorooctaneseleninic acid (C_8_F_17_Se(O)OH) was employed as the catalyst in allylic oxidations leading to α,β-unsaturated carbonyl compounds in moderate to good yield. After a reductive workup with sodium metabisulfite the catalyst was recovered by fluorous extraction in the form of bis(perfluorooctyl) diselenide, which itself serves as a convenient catalyst precursor [141]. The same oxidation system was used for the efficient oxidation of alkyl aryl ketones to ketoacids and even benzylic methylene groups were oxidized to the corresponding ketones [142]. Polystyrene-supported benzeneseleninic acid and hydrogen peroxide was shown to be an efficient and mild reagent for the directly conversion of both aromatic and aliphatic aldoximes into carboxylic acid esters [143].

**Scheme 23:**
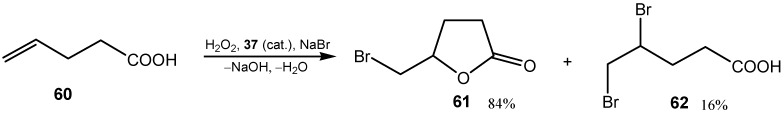
Bromolactonization of an γ,δ-unsaturated acid via benzeneseleninic acid catalyzed oxidation of NaBr with H_2_O_2_.

Areneseleninic acids like selenoxides were used as catalysts for the oxidation of bromide with hydrogen peroxide to hypobromite and bromine in a two-phase reaction mixture. Among various areneseleninic acids tested as catalysts, the most effective were benzeneseleninic acid **51** and 4-methoxybenzeneseleninic acid. Br_2_ and NaOBr generated *in situ* bring on the cyclization of γ,δ-unsaturated acids, such as for example, 4-pentenoic acid **60** or related unsaturated alcohols, which give the lactone **61** accompanied by a small amount of dibromo acid **62** (Scheme 23). Similarly, the electrophilic bromination of activated aromatic rings can be performed in high yield [144,145].

**Scheme 24:**
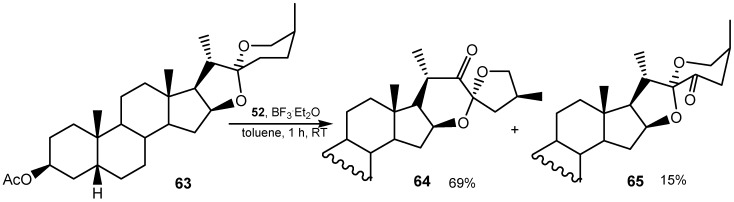
Reaction of sarsasapogenin acetate **63** with benzeneseleninic anhydride.

Oxidation of phenols with anhydride **52** was applied for conversion of the phenolic part of chiral cyclophanes into quinone [146]. Treatment of 13-ketobaccatin III (a precursor of the anticancer drug pactitaxel) with the same reagent resulted in novel A, B ring rearranged products [147]. 1,2-Dicarbonyl compounds employed as key-intermediates in indolone-*N*-oxide synthesis were prepared in 20%–70% yield by direct oxidation of aryl- and alkyl-substituted alkenes by benzeneseleninic anhydride [148] *t-*Butylhydroperoxide in the presence of catalytic amount of benzeneseleninic anhydride was an effective oxidizing agent for the selective oxidation of alcohols at the benzylic position. The ketones were obtained in good yields [149]. Reaction of 2,3-dioxochlorins with benzeneselenic anhydride results in the formation of unusual ring-contracted azetine derivatives that further react with anhydride to afford porpholactones [150]. Direct dehydrogenation of spirostan sapogenin **63** with benzeneseleninic anhydride/iodoxybenzene in the presence of BF_3_/Et_2_O, afforded 23-oxosapogenins in addition to their 22-oxo-23-spiro isomers. In the case of sarsasapogenin acetate **63** the major reaction product the 23-spiro-22-ketone **64** was accompanied by 23-oxosarsasapogenin acetate **65** (Scheme 24) [151].

Most recently it has been revealed, that the cyclic seleninate ester **8** acts as a catalyst for the rapid and chemoselective oxidations of sulfides to sulfoxides with hydrogen peroxide in the presence of trifluoroacetic acid and also act as a catalyst for the conversions of alkenes to epoxides and of morpholinyl enamines to α-hydroxyketones. In some cases, such as in the oxidations of styrene, α-methylstyrene, and cinnamyl alcohol, oxidative cleavage of the alkene instead of epoxidation occurred to give either benzaldehyde or acetophenone. α-Methylstyrene oxide was converted to acetophenone under the reaction conditions, while α-methylstyrenediol did not react. Oxidations of morpholinyl enamines proceeds by the initial formation of diaminodioxanes, which are hydrolyzed *in situ* to give α-hydroxyketones such as 2-hydroxycyclohexanone [152].

### 3.3. Diselenides

Diselenides RSeSeR are known as precatalysts for catalytic oxidations. The use of these widely available compounds in the past decades as catalysts for the oxidation of different functional groups of organic compounds has been summarized elsewhere, e.g., in the reviews [4,6,7,9,17,146,153]. Diphenyl diselenide is a commercially available compound, whereas other diselenides can be easily obtained in the reaction of alkyl, aryl, and heteroaryl halides or tosylates with dilithium or disodium diselenide formed *in situ* from elemental alkaline metal and selenium in aprotic media [7,146,147,153,154].

**Scheme 25:**
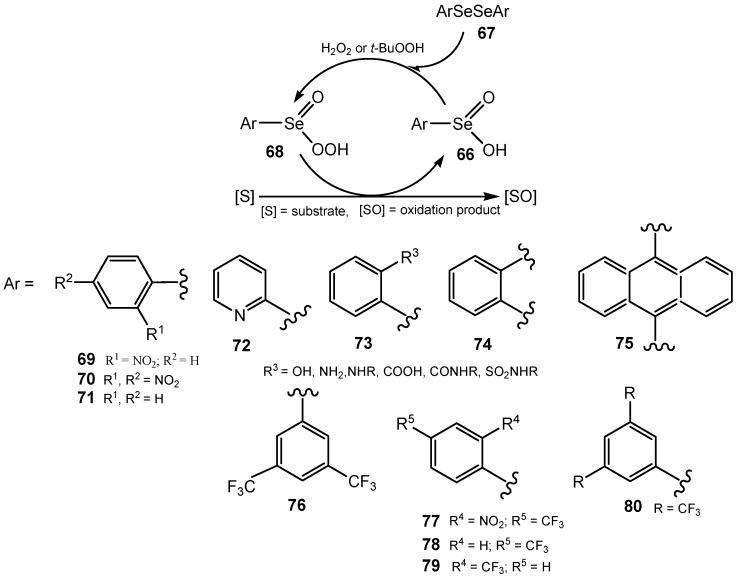
The mechanism of hydroperoxide oxidation of organic substrate catalyzed by diaryl diselenide or areneseleninic acid.

Currently, diselenides have been used more frequently than seleninic acids. They act as catalysts for the oxidation of different organic compounds with hydrogen peroxide, TBHP and other oxygen donors. The proposed mechanism of the oxidation of organic substrate in the presence of areneseleninic acid **66** or its precursor the diaryl diselenide **67**, is presented in Scheme 25. Diselenide **67** is oxidized *in situ* with hydrogen peroxide or TBHP into seleninic acid **66** and then to active oxygen donor the areneperoxyseleninic acid **68**. 2-Nitro and 2,4-dinitrobenzeneperoxyseleninic acids were obtained by hydrogen peroxide oxidation of the corresponding diaryl diselenides and fully characterized [155]. In an anhydrous medium the mechanism can be more complex. The well-known oxidation of activated alcohols (e.g., benzyl alcohol) with TBHP catalyzed by diphenyl diselenide was reinvestigated using a range of analytical techniques. 

**Scheme 26:**
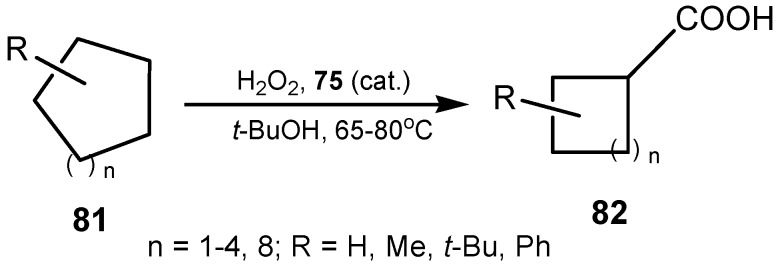
Oxidative conversion of cycloalkanones **81** into cycloalkanecarboxylic acids **82** catalyzed by poly(bis-9,10-anthracenylene) diselenide **75**.

Evidence was found for the involvement of seleninic anhydride in the catalytic mechanism. An improved protocol for the selective oxidation of activated alcohols to aldehydes was devised resulting in significantly decreased catalyst loadings (<1%) [156]. It has been observed that the effectiveness of selenium catalysts strongly depends on the substrate used. While *ortho*-substituted diphenyl diselenides are the best catalysts for hydrogen peroxide oxidation of sulfides into sulfoxides and ketazines to their parent ketones, the poly(bis-1,2-phenylene) diselenide **74** was selected for preparative oxidation of various aromatic aldazines, aldoximes, and conversion of tosylhydrazones into arenecarboxylic acids [157]. In the presence of poly(bis-9,10-anthracenylene) diselenide (**75**) a broad spectrum of aliphatic, unsaturated and aromatic nitriles was obtained, in excellent preparative yields, by oxidation of the corresponding *N*,*N-*dimethylhydrazones [158]. It was the catalyst of choice for oxidation of cycloalkanones **81** to cycloalkanecarboxylic acids **82** (Scheme 26). Since the cycloalkanones are cheap and easily available substrates, the elaborated method is suitable for the synthesis of acids **82**, particularly those having five-, six- and seven-membered rings. The mechanism of the ring contraction was also proposed [159].

The bis[2-nitro-4-(trifluoromethyl)phenyl] diselenide **77** was found to be an efficient catalyst for hydrogen peroxide oxidative degradation of the electron-rich benzene ring of phenols. 

**Scheme 27:**
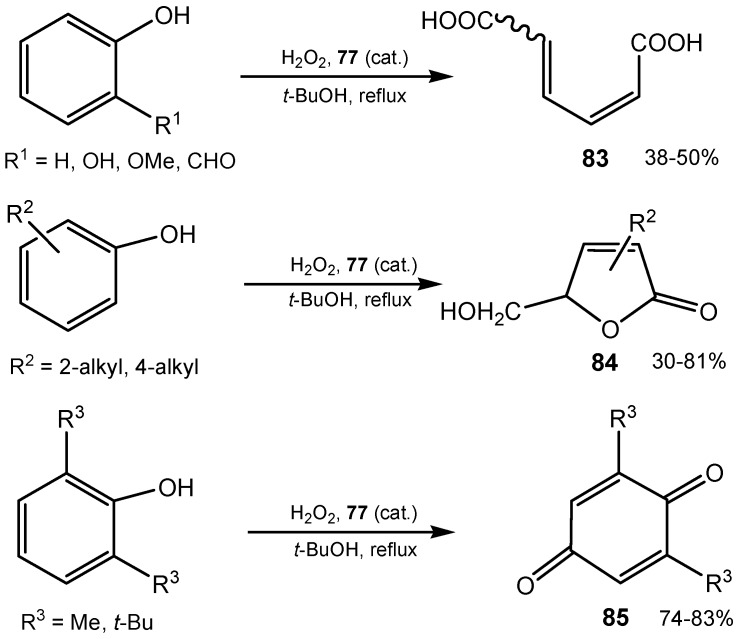
Oxidative conversion of phenols to muconic acid and muconolactones.

Depending on the substrate used, muconic acid ((2*E*,4*E*)-hexa-2,4-dienedioic acid, **83**), muconolactones **84** or 1,4-benzoquinones **85** were produced in satisfactory to good yields (Scheme 27). Similar ring-degradation took place, when substituted naphthalenes were oxidized. Cinnamic acid or benzofurane derivatives were the final products [160,161].

The 1,2-bis[3,5-bis(trifluoromethyl)phenyl] diselenide **80** has been reported as significantly more active than other previously described selenium catalysts for the epoxidation and Baeyer-Villiger oxidation of carbonyl compounds with hydrogen peroxide [162,163]. Most recently dibenzyl diselenide was found to be the best precatalyst for the same oxidation of (*E*)-α,β-unsaturated ketones with hydrogen peroxide. The catalyst used in this reaction could be recycled and reused several times [164].

A highly efficient and green strategy for the epoxidation of fatty esters, combining a green oxidant (aq. hydrogen peroxide) and a recyclable catalyst **80** was presented. The possibility of integrating renewable solvents derived from glycerol in the productive cycle of biodiesel commodities was also explored. Fluorinated solvents (both commercial and glycerol-derived) play a double key role on this methodology. They strongly accelerate epoxidation reaction with respect to common non-fluorinated solvents and, on the other hand, some of them allow catalyst recycling [165].

It was reported that diselenides bearing trifluoromethanesulfonate groups catalyzed the oxidation of cyclohexanones into the corresponding lactones in 59%–99% yield. The oxidation of 2,5-dimethoxybenzaldehyde made it possible to obtain of 2,5-dimethoxyphenol almost quantitatively. The reaction was carried out in dichloromethane at room temperature and no fluorous solvent was required [166]. A low loading and recyclable diselenide **80** was found as an excellent catalyst for hydrogen peroxide oxidation of cyclohexene to *trans*-1,2-cyclohexanediol in 96% yield [167]. 

**Scheme 28:**
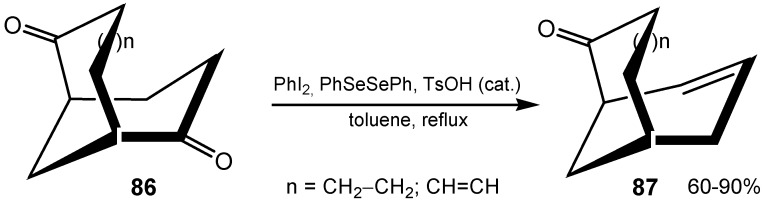
Oxidative unsaturation of (+)-(1*S*,5*S*)-bicyclo[3.3.1]-nonane-2,6-diones.

Novel optically active diselenides, having a chiral oxazoline moiety, were prepared and used as catalysts for hydrogen peroxide oxidation of a variety of cyclobutanones. The corresponding γ-lactones were obtained in up to 92% yield but the enantioselectivity of the product was not satisfactory [168]. The enantiospecific synthesis of several bicyclic enones **87**, starting from enantiomerically pure (+)-(1*S*,5*S*)-bicyclo[3.3.1]-nonane-2,6-diones **86** includes an oxidative unsaturation step with PhIO_2_/ (PhSe)_2_ (Scheme 28) [169].

Some diaryl diselenides, patricularly diphenyl diselenide and di(3-fluorophenyl)diselenide) are effective and reusable catalysts for the hydrogen peroxide dehydration of oximes leading to a practical and scalable preparation of useful nitriles under mild conditions [170].

A two-stage catalytic process using H_2_O_2_/urea as a primary oxidant and benzothiazine dioxide with diselenide **80** as oxygen transfer cocatalysts was applied to the epoxidation of alkenes and oxidation of saturated and unsaturated aliphatic substrates. For tertiary alkanes and cycloalkanes C-H hydroxylation is strongly preferred, even for starting materials in which methylene oxidation enjoys a significant statistical advantage. Substrates possessing equatorial C-H groups on cyclohexane rings are optimal, as highlighted by the reaction of *cis*-decalin. Alkenes are efficiently oxidized to epoxides [171]. Hydrogen peroxide in the presence of diphenyl diselenide oxidized alkenes to epoxides that are subsequently hydrolyzed to 1,2-diols [172].

**Scheme 29:**
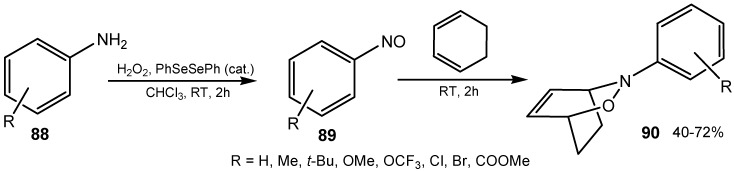
Organoselenium-catalyzed oxidation of aniline analogs followed by hetero-Diels-Alder reaction with 1,3-cyclohexadiene.

A method for *in situ* generation of nitroso compounds from oxidation of anilines **88** by hydrogen peroxide in the presence diphenyl diselenide as catalyst was developed. The generated nitroso compounds **89** were subsequently used in hetero-Diels-Alder reactions. A variety of oxazines **90** were synthesized in reasonable to good yields by this one-pot procedure using primary aromatic amines with different substituents and various conjugated dienes (Scheme 29) [173].

Water-soluble diphenyl diselenides having the benzene ring substituted with the *N*-methyl-imidazolium group, have proven to be efficient catalysts for the oxidation of NaBr with H_2_O_2_, and various organic substrates can be brominated in this way [174].

### 3.4. Selenenamides and Related Compounds 

**Figure 1:**
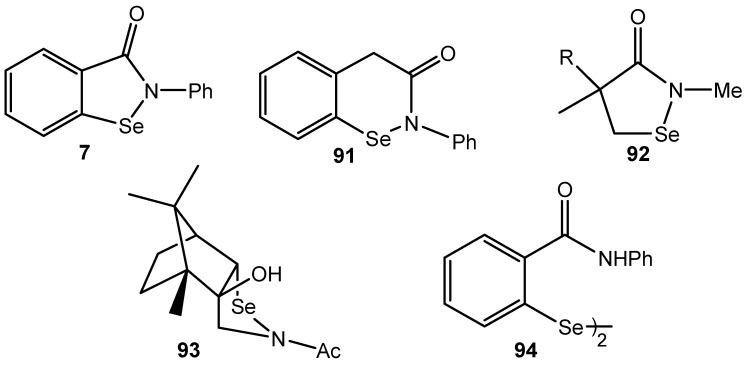
The cyclic selenenamides **7**, **91**–**93** and related diselenide **94**.

Three decades ago it was revealed that a simple, synthetically available cyclic selenenamide 2-phenylbenzisoselenazol-3(2*H*)-one (**7**) named ebselen could act against oxidative stress in a similar way to the common selenoenzyme glutathione peroxidase (GPx). Later it was found that other 2-substituted benzisoselenazol-3(2*H*)-ones, cyclic selenenamides **91**–**93** and their open-chain analogues, among them bis[(2-carbamoylphenyl)-phenyl] diselenide (**94**, Figure 1) are able to deactivate active oxygen species present in the living cell, such as peroxides, hydroperoxides, hydroxyl radicals and superoxide anion. The mode of their biological action has been postulated to be similar to that observed for GPx, and results in dehydrogenation of thiols into disulfides while hydrogen peroxide is reduced to water. Biomimetic oxidation of various thiols into disulfides, moderate by ebselen and the other organoselenium compounds, is beyond the scope of this article and it has been discussed elsewhere [175,176,177,178,179].

Other works showed the evidence that the ebselen, related selenenamides and diselenides could catalyze the oxidation of various organic compounds with hydroperoxides. The catalyst **7** (R=H) was used in 5 mol %, and diselenide **94** in 2.5 mol % while the stoichiometric oxidant was 30% hydrogen peroxide or 80% TBHP.

**Scheme 30:**
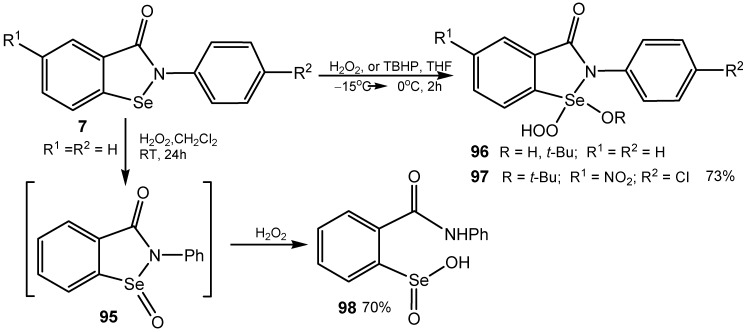
Oxidation of benzisoselenazol-3(2*H*)-ones with hydroperoxides.

The catalytic acivity of ebselen (**7**, R = Ph) for hydroperoxide oxidation of different groups of organic compounds, raised a question about the role of the oxidant and catalyst in this reaction, the more so because a similar activity of seleninamide (selenoxide) **95**, and related open-chain diselenides **94** was observed [180,181]. Oxidation of aromatic aldehydes, having electron-donating substituents, with TBHP in the presence of ebselen led almost exclusively to the corresponding carboxylic acids, thus avoiding the Baeyer-Villiger rearrangement. Studying this reaction in more detail, it was found that ebselen treated with a large excess of hydrogen peroxide, under cooling, yielded an unstable crystalline compound, *i.e*., the hydroperoxyselenurane **96**. A more stable and fully characterized analogue **97** was obtained under similar conditions by oxidation of the corresponding benzisoselenazol-3(2*H*)-one with hydrogen peroxide or with TBHP (Scheme 30). It seems possible, that treatment of organic substrates in the presence of ebselen, but also seleninamide **95** or related diselenide, with a large amount (100-fold molar excess) of hydroperoxide results in the formation of hydroperoxyselenurane **96**, being the active oxygen donor involved in oxidation of the organic substrate [182]. On the other hand, it has been shown that the GPx-like catalytic mechanism of ebselen is different for the antioxidant and anti-inflammatory activities and involves reversible cyclization of the selenenic acid (RSeOH) to ebselen. The long duration reaction of ebselen with hydrogen peroxide (10-fold molar excess) produces exclusively the corresponding seleninic acid **98**, being a crucial intermediate involved in the postulated oxidation mechanism [183].

Using H_2_O_2_/ebselen sulfides were exclusively oxidized into sulfoxides. Aromatic aldoximes oxidized in methanol give carboxymethyl esters in 62%–82% yield. Nitriles were produced, almost quatitatively, from *N*,*N*-dimethylhydrazones by oxidation with H_2_O_2_/ebselen or from benzylamines, oxidized with TBHP/ebselen in 62%–70% yield. Hydrogen peroxide oxidation of ketazines gave the parent ketones in 62%–98% yield. Cyclooctene treated with TBHP gave epoxide, accompanied with trace amounts of 3-hydroxycyclooctene, resulting from α-hydroxylation. It was postulated, that all these reactions have ionic character [6,9,17].

**Scheme 31:**
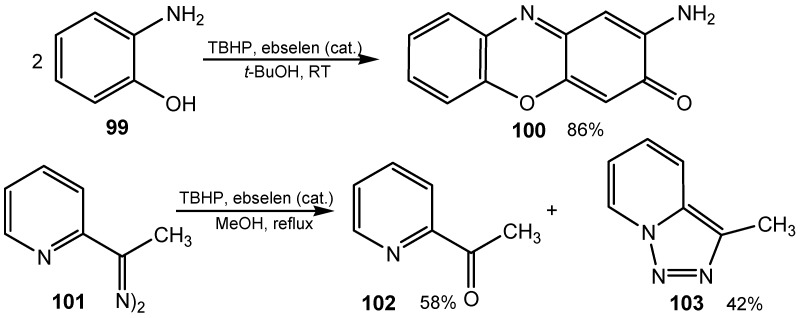
TBHP/ebselen-mediated oxidation of *o*-aminophenol **99** and ketazine **101**.

Another mechanism, involving a radical could also be envisaged to explain some other reactions. Catalyzed by ebselen, TBHP oxidation of alkylarenes to alkyl aryl ketones [184], anthracene to antraquinone, 1,4-dimethoxyarenes to 1,4-quinones (e.g., 2-methyl-1,4-dimethoxynaphthalene to menaquinone) [185], and the oxidative coupling of 2-aminophenol **99** to phenoxazinone **100** gave results similar to those with the one-electron oxidants Ce(IV), Ag(II), or Mn(III) [180,181], Moreover, oxidation of ketazine **101**, derived from 2-acetylpyridine, gave a mixture of ketone **102** and condensed triazole **103** (Scheme 31) [184], The same results were obtained when cerium ammonium nitrate was used as the reagent. This suggest, that the reaction proceeds via cation-radicals.

The catalytic activity of the various isoselenazolones in the bromolactonization of pent-4-enoic acid was investigated. Isoselenazolone **104** was found as an efficient catalyst in three reactions: the bromolactonization of alkenoic acids with bromine or *N-*bromosuccinimide (NBS) in the presence of potassium carbonate as base, the bromoesterification of alkenes using a variety of carboxylic acids, and the oxidation of secondary alcohols to ketones using bromine as an oxidizing agent [186].

**Figure 2:**
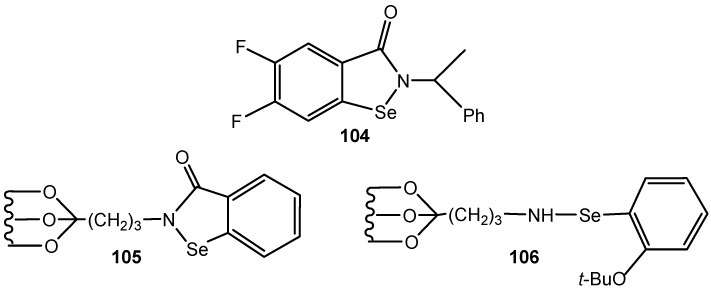
Isoselenazolone **104**, immobilized ebselen **105** and selenenamide **106**.

Some benzisoselenazol-3(2*H*)-ones and open-chain selenenamide were covalently immobilized to the solid support, either silica or polymer. Two of them **105** and **106** (Figure 2) exhibited appreciable catalytic activity similar to the activity of ebselen, and could be easily recovered by filtration, and reused. The catalyst **105** has been applied for hydrogen peroxide oxidation of sulfides and TBHP oxidation of the aromatic aldehydes to acids, and alkylarenes to alkyl aryl ketones [187].The catalytic activity of **106** was demonstrated in TBHP oxidation of aldehydes to the corresponding carboxylic acids and benzylamines to nitriles. Moreover, the process was employed for hydrogen peroxide oxidation of azomethine compounds such as tosylhydrazones to the corresponding arenecarboxylic acids and ketones. Oximes were oxidized to the mixtures of esters and carboxylic acids while the *N*,*N*-dimethylhydrazones produced the mixtures of nitriles and carboxylic acids, depending on the substrate used and the reaction conditions [188].

**Scheme 32:**
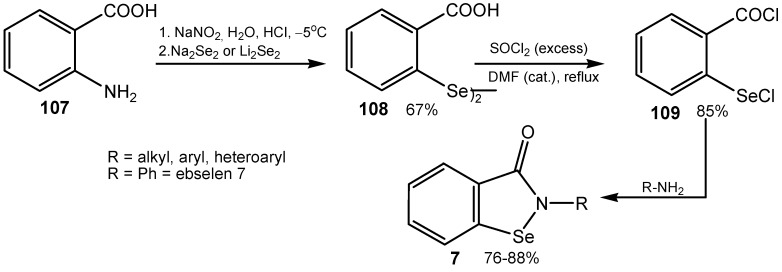
A general method for synthesis of benzisoselenazol-3(2*H*)-ones.

It should be noted that ebselen and its analogs **7** can be obtained easily, in the four-step synthesis, from anthranilic acid **107** and aniline via bis(2-carboxyphenyl) diselenide **108** and dichloride **109** (Scheme 32). The method has a more general value, because by using of various amines and other compounds with primary amino groups, different benzisoselenazol-3(2*H*)-ones and 2-substituted diphenyl diselenides, also these bounded to solid support, can be obtained in high yields [189,190]. More recently other competitive methods for the synthesis of ebselen and other benzisoselenazolones have been elaborated [9,90,186,191,192].

## 4. Conclusions

In this work an attempt has been made to summarize the progress in the exploitation of the selenium compounds as oxidants and oxygen-transfer agents. It was shown that the classic reagent SeO_2_ is still used in modern organic synthesis as a primary oxidant or as catalyst. The biggest progress has been observed in the design and application of organoselenium compounds, particularly diselenides, areneseleninic acids, their derivatives and cyclic selenenamides as catalysts for oxidation of different functional groups of organic compounds with hydrogen peroxide, *t*-butyl hydroperoxide and iodoxybenzene. The most important reactions have been allylic α-hydroxylation, α-oxygenation of alkenes and enolizable ketones, epoxydation of alkenes, oxidation of methyl groups in arenes and heteroarenes, 1,2-hydroxylation of alkenes, dehydrogenation and oxidative C-O and C-C cleavage, oxidative ring closure and ring transformations, heteroatom *N*- and *S*-oxidation, Baeyer-Villiger conversion of ketones into lactones, regeneration of carbonyl groups from azomethine groups and others. The mechanisms of some important reactions have been discussed and their scope and limitations have been indicated. Links have been provided to reviews summarizing the earlier literature and to the methods of preparation of organoselenium catalysts. We expect that this review will serve as a valuable critical overview of the area, and it is hoped that the contribution will helps encourage further research in this field.

## References

[1] Paulmier C. (1986). Selenium Reagents and Intermediates in Organic Synthesis.

[2] Back T.G., Patai S. (1987). Preparative uses of organoselenium and organotellurium compounds. The Chemistry of Organic Selenium and Tellurium Compounds.

[3] Młochowski J. (1998). Organoselenium compounds as oxidants and oxidation catalysts. Phosphorus Sulphur Silicon.

[4] Back T.G., Back T.G. (1999). Oxidation with Selenium Reagents. Organoselenium Chemistry. A Practical Approach.

[5] Nishibayashi Y., Uemura S. (2000). Selenium Compounds as Ligands and Catalysts. Top. Curr. Chem..

[6] Młochowski J., Brząszcz M., Giurg M., Palus J., Wójtowicz H. (2003). Selenium-Promoted Oxidation of Organic Compounds: Reactions and Mechanisms. Eur. J. Org. Chem..

[7] Giurg M., Syper S. (2008). Diaryl Diselenides and Related Compounds as Oxygen-Transfer Agents. Phosphorus Sulphur Silicon.

[8] Freudendahl D.M., Santoro S., Shahzad S.A., Santi C., Wirth T. (2009). Green Chemistry with Selenium Reagents: Development of Efficient Catalytic Reactions. Angew. Chem..

[9] Młochowski J., Lisiak R., Wójtowicz-Młochowska H., Patai S., Rappoport Z., Liebman J.F., Marek I. (2012). Organoselenium and organotellurium oxidation and reduction. The Chemistry of Organic Selenium and Tellurium Compounds.

[10] Nomoto A., Ogawa A., Patai S., Rappoport Z., Liebman J.F., Marek I. (2012). Preparative uses of organoselenium and organotellurium compounds. The Chemistry of Organic Selenium and Tellurium Compounds.

[11] Sheldon R.A. (1993). Homogenous and heterogenous catalytic oxidations with peroxide reagents. Top. Curr. Chem..

[12] Młochowski J., Said S.B. (1997). Catalyzed Hydrogen Peroxide Oxidation of Organic Compounds. Pol. J. Chem..

[13] Sanderson W.F. (2000). Cleaner industrial processes using hydrogen peroxide. Pure Appl. Chem..

[14] Ten Brink G.-J., Arends I.W.C.E., Sheldon R.A. (2004). The Bayer-Villiger Reaction: New developments toward greener procedures. Chem. Rev..

[15] Chen M.S., White M.C. (2007). A predictably selective aliphatic C-H oxidation reactions for complex molecule synthesis. Science.

[16] Gelalcha F.G. (2007). Heterocyclic peroxides in selective oxidations. Chem. Rev..

[17] Młochowski J., Peczyńska-Czoch W., Piętka-Ottlik M., Wójtowicz-Młochowska H. (2011). Non-Metal and Enzymatic Catalysts for Hydroperoxide Oxidation of Organic Compounds. Open Catal. J..

[18] Breder A., Ortgies S. (2015). Recent developments in sulphur- and selenium-catalysed oxidative and isophysic functionalization reaction of alkenes. Tetrahedron Lett..

[19] Lattanzi A., Knohel P., Molander G.A. (2014). Oxidation of sulfur, selenium and tellurium. Comprehensive Organic Synthesis.

[20] Nogueira C.W., Rocha J.B.T., Patai S., Rappoport Z., Liebman J.F., Marek I. (2012). Organoselenium and organotellurium compounds: Toxicology and pharmacology. The Chemistry of Organic Selenium and Tellurium Compounds.

[21] Nogueira C.W., Zeni G., Rocha J.T.B. (2004). Organoselenium and Organotellurium Compounds: Toxicology and Pharmacology. Chem. Rev..

[22] Nogueira C.W., Rocha J.T.B. (2010). Diphenyl diselenide a janus-faced molecule. J. Braz. Chem. Soc..

[23] Meotti F.C., Borges V.C., Zeni G., Rocha J.B.T., Nogueira C.W. (2003). Potential renal and hepatic toxicity of diphenyl diselenide, diphenyl ditelluride and Ebselen for rats and mice. Toxicol. Lett..

[24] Santoro S., Azaredo J.B., Nascimento V., Braga A.L., Sancineto L., Santi C. (2014). The green side of the moon: Eco-friendly aspects of organoselenium chemistry. RCS Adv..

[25] Braga A.L., Schwab R.S., Rodrigues O.E.D., Santi C. (2014). Organoselenium Chemistry: Between Synthesis and Biochemistry.

[26] Santi C., Santoro S., Battiselli B. (2010). Organoselenium Compounds as Catalysts in Nature and Laboratory. Curr. Org. Chem..

[27] Riley H.L., Morley J.F., Friend N.A.C. (1932). Selenium dioxide, a new oxidizing agent. Part I. Its reaction with aldehydes and ketones. J. Chem. Soc..

[28] Maity A.C. (2008). Selenium Dioxide (SeO_2_)—A versatile reagent. Synlett.

[29] McNally J., Fuchs P.L. (2010). Selenium(IV) oxide—*tert*-Butylhydroperoxide. e-EROS Encyclopedia of Reagents for Organic Synthesis.

[30] Hoekstra W.J., Fuchs P.L. (2010). Selenium(IV) oxide. e-EROS Encyclopedia of Reagents for Organic Synthesis.

[31] Gebhardt C., Priewisch B., Ivran E., Rueck-Braun K. (2008). Oxidation of anilines with hydrogen peroxide and selenium dioxide as catalyst. Synthesis.

[32] Ogawa A., Yamamoto Y., Oshima K. (2004). Selenium and tellurium in organic synthesis. Main Group Metals in Organic Synthesis.

[33] Sharpless K.B., Lauer R.F. (1972). Selenium dioxide oxidation of olefins. Evidence for the intermediacy of allylseleninic acids. J. Am. Chem. Soc..

[34] Singelton D.A., Hang C. (2000). Isotope Effects and the Mechanism of Allylic Hydroxylation of Alkenes with Selenium Dioxide. J. Org. Chem..

[35] Ra C.S., Park G. (2003). Ab initio studies of the allylic hydroxylation: DFT calculation on the reaction of 2-methyl-2-butene with selenium dioxide Tetrahedron. Lett..

[36] Park G., Hwang J.C., Jung W.S., Ra C.S. (2005). Stereochemical Course of the Allylic Hydroxylation: Reaction of 1-*tert*-Butyl-4-alkylidenecyclohexanes with Selenium Dioxide. Bull. Korean. Chem. Soc..

[37] Patel R.M., Puranik V.G., Argade N.P. (2011). Regio- and stereoselective selenium dioxide allylic oxidation of (*E*)-dialkyl alkylidenesuccinates to (*Z*)-allylic alcohols: Synthesis of natural and unnatural butenolides. Org. Biomol. Chem..

[38] Khurana J.M., Dawra K., Majumdar S. (2009). An efficient 1,3-allylic carbonyl transposition of chalcones. Monatsh. Chem..

[39] Strommer R., Straus W., Emmert H., Sailer R., Steiner E., Resinger E.W., Haslinger E., Schramm H.W. (2001). Synthesis and biological activity of oxidation products of the antiprogestine mifepristone. Monatsh. Chem..

[40] Strommer R., Hoedl C., Strauss W., Sailer R., Haslinger E., Schramm H.W., Seger C. (2004). Synthesis of 6-hydroxy derivatives of steroidal hormones by SeO_2_ mediated oxidation. Monatsh. Chem..

[41] Kim H.S., Kang J.H. (2001). Selenium Dioxide Oxidation of 3β-Benzoyloxy-5α-cholest-8(14)-en-15-one: Chemical Synthesis of 3β-Hydroxy-5α-cholest-8(14),16-dien-15-one. Bull. Korean. Chem. Soc..

[42] Ma E., Choi T. (2009). An Efficient 4β-Hydroxylation of Steroidal 5-en-3β-ols and 1,4-Conjugation of Steroidal 4-en-3-ones Using SeO_2_ Oxidation. Bull. Korean. Chem. Soc..

[43] Czajkowska D., Morzycki J.W., Santillan R., Siergiejczyk L. (2009). Synthesis of ‘glycospirostanes’ via ring-closing metathesis. Steroids.

[44] Fujioka H., Kotoku N., Sawama Y., Nagatomi Y., Yasuyuki Kita Y. (2002). Concise asymmetric synthesis of a model compound, (4*S*,5*S*,6*S*)-6-(2,2-dimethoxy)ethyl-4,5-epoxy-6-hydroxy-2-cyclohexenone, for the cyclohexenone core of scyphostatin. Tetrahedron Lett..

[45] Mushfiq M., Rehman S.R. (2010). One-pot SeO_2_ oxidation of steroidal alkenes. Oxidation Commun..

[46] Kharitonov Y.V., Shults E.E., Gatilov Y.V., Bagryanskaya I.Y., Shakirov M.M., Tolstikov G.A. (2012). Synthetic transformations of higher terpenoids. XXVII. Synthesis of 7-hydroxylabdanoids and their transformations. Chem. Nat. Compd..

[47] Ernet T., Haufe G. (1997). Allylic hydroxylation of vinyl fluorides. Synthesis.

[48] Fairlamb I.J.S., Dickinson J.M., Pegg M. (2001). Selenium dioxide *E*-methyl oxidation of suitably protected geranyl derivatives—Synthesis of farnesyl mimics. Tetrahedron Lett..

[49] Smith A.B., Bosanac T., Basu K. (2009). Evolution of the Total Synthesis of (−)-Okilactomycin Exploiting a Tandem Oxy-Cope Rearrangement/Oxidation, a Petasis−Ferrier Union/Rearrangement, and Ring-Closing Metathesis. J. Am. Chem. Soc..

[50] Huang Q., Pennington J.D., Williams H.J., Scott A.I. (2006). Models for Taxol Biosynthesis: SeO_2_ Oxidation of Taxadiene. Synth. Commun..

[51] Manktala R., Dhillon R.S., Chhabra B.R. (2006). Urea-hydrogen peroxide and microwave. An eco-friendly blend for allylic oxidation of alkenes with catalytic selenium dioxide. Indian J. Chem. Sect. B.

[52] Sabir H., Mukta S. (2010). Selective oxygenation and plant-growth regulatory activity of sesquiterpene lactones. J. Phys. Sci..

[53] Sabir H., Mukta S., Meenakshi H. (2010). Highly selective oxygenations of olefins over selenium dioxide using urea hydrogen peroxide as oxidising agent with sesquiterpene lactones. J. Glob. Pharma Technol..

[54] Barrero A.F., Quildez Del Moral J.F., Del Mar Herador M., Sanchez E.M., Arteaga J.F. (2006). Regio- and Enantioselective Functionalization of Acyclic Polyprenoids. J. Mex. Chem. Soc..

[55] Warpehoski M.A., Chabaud B., Sharpless K.B. (1982). Selenium dioxide oxidation of endocyclic olefins. Evidence for a dissociation-recombination pathway. J. Org. Chem..

[56] Aranda G., Bertranne-Delahaye M., Azerad R., Maurs M., Cortés M., Ramirez H., Vernal G., Prangé T. (1997). Practical and Efficient 1α-Hydroxylation of 4,4-Dimethyl-2-Ene Derivatives in Terpenic Series. Synth. Commun..

[57] Chabaud B., Sharpless K.B. (1979). Oxidation of acetylenes with *tert*-butyl hydroperoxide catalyzed by selenium dioxide. J. Org. Chem..

[58] Gogoi P., Sharma S.D., Konwar D. (2007). SeO_2_/H_2_O_2_/H_2_O-Dioxane: A new catalytic system for *trans* dihydroxylation of olefins. Lett. Org. Chem..

[59] Chang M.Y., Lin C.H., Chen Y.L. (2010). Selenium dioxide–mediated methoxyhydroxylation of cyclic arylolefin. Tetrahedron Lett..

[60] Chang M.Y., Hsu R.T., Cheng H.P., Lin P.J. (2006). Concise synthesis of 3-arylpiperidines. Heterocycles.

[61] Knothe G., Glass R.S., Schroeder T.B., Bagby M.O., Weisleder D. (1997). Reaction of isolated double bonds with selenium dioxide/hydrogen peroxide: Formation of novel selenite esters. Synthesis.

[62] Goswami S., Mukherjee R., Mukherjee R., Jana S., Maity A.C., Adak A. (2005). Simple and EfficientSynthesis of 2,7-Difunctionalized-1,8-Naphthyridines. Molecules.

[63] Abd El-Monem Y.H.A., Elkanzi N.A., Mohamed N.M.M. (2013). Synthesis of some new spirocyclic β-lactams and spirocyclic thiazolidin-4-one derivatives. Eur. J. Chem..

[64] Rakhimov A.I., Shul’man R.B., Fedunov R.G. (2011). Oxidation of 6-methyl-2,4-dioxypyrimidine with selenious acid. Russ. J. Gen. Chem..

[65] Goswami S., Adak A.K. (2003). Microwave Assisted Improved Synthesis of 6-Formylpterin and Other Heterocyclic Mono- and Di-aldehydes. Synth. Commun..

[66] Guo Y., Yan Y.-Y., Yang C., Yu X., Zhi X.Y., Xu H. (2012). Regioselective synthesis of fraxinellone-based hydrazone derivatives as insecticidal agents. Bioorg. Med. Chem. Lett..

[67] Bobrov D.N., Tyvorskii V.I. (2010). Facile synthesis of caerulomycin E by the formation of 2,2ʹ-bipyridine core via a 2-pyridyl substituted 4*H*-pyran-4-one. Formal synthesis of caerulomycin A. Tetrahedron.

[68] Hannesian S., Szychowski J., Maianti J.P. (2009). Synthesis and Comparative Antibacterial Activity of Verdamicin C2 and C2a. A New Oxidation of Primary Allylic Azides in Dihydro[2*H*]pyrans. Org. Lett..

[69] Chandrasekhar R., Gopalan B., Nanjan M.J. (2011). Synthesis and Characterisation of 3-Hydroxy-4, 5-dihydro[1,2,3] Oxadiazolo [3,4-A]Quinolin-10-ium and its Fluoro derivative. Int. J. ChemTech Res..

[70] Tagawa Y., Yamashita K., Higuchi Y., Goto Y. (2003). Improved Oxidation of Active Methyl Group of *N*-Heteroaromatic Compounds by Selenium Dioxide in the Presence of *tert*-Butyl Hydroperoxide. Heterocycles.

[71] Goswami S., Maity A.C. (2007). Oxidative Removal of Heterocyclic Alkyl or Sugar Side Chain by Microwave: A Simple Step to Xanthopterin, 6-Formylpterin, and 3-Hydroxymethyl-2(1*H*)-Quinoxalinone. Chem. Lett..

[72] Habibi M., Bayat Y., Marandi R., Mehrdadsharif A.A., Salahi S. (2012). One-pot synthesis of 4*H*-pyran-4-one carboxaldehyde derivatives by using selenium dioxide as a reusable oxidant. Asian J. Chem..

[73] Remias J.E., Sen A. (2003). Nitrogen oxides/selenium dioxide-mediated benzylic oxidations. J. Mol. Cat. A Chem..

[74] Jordan J.A., Gribble G.W., Badenock J.C. (2011). A concise total synthesis of bruceolline E. Tetrahedron Lett..

[75] Sivaperuman S., Santhanagopalan P., Amali I.B., Shanmugam M. (2009). Chemoselective Selenium Dioxide Oxidation of 1,4-Adducts Derived from Substituted Arylidene Acetophenones. Synth. Commun..

[76] Mehta G., Shinde H.M. (2003). Enantiospecific total synthesis of 6-epi-(−)-hamigeran B. IntramolecularHeck reaction in a sterically constrained environment. Tetrahedron Lett..

[77] Young R.M., Davies-Coleman M.T. (2011). Microwave-assisted selenium dioxide oxidation of aryl methyl ketones to aryl glyoxals. Tetrahedron Lett..

[78] Shirude S.T., Patel P., Giridhar R., Yadav M.R. (2006). An efficient and time saving microwave- assisted selenium dioxide oxidation of 1,2-diarylethanones. Indian J. Chem. Sect. B.

[79] Belsey S., Danks T.N., Wagner G. (2006). Microwave-Assisted Selenium Dioxide Oxidation of Camphor Derivatives to α-Dicarbonyl Compounds and Oxoimines. Synth. Commun..

[80] Taherpour A., Kamal S.B. (2007). 1,2,3-trione compounds synthesis by oxidation 1,3- diketones. Asian J. Chem..

[81] Xiao-Hua C., Hai-Jun Y., Guo-Lin Z. (2005). Aromatization of 1,4-dihydropyridines with selenium dioxide. Can. J. Chem..

[82] Paul S., Shivani S., Gupta M., Choudhary D., Gupta R. (2007). Oxidative Aromatization of Hantzsch 1,4-Dihydropyridines by SiO_2_/P_2_O_5_-SeO_2_ under Mild and Heterogeneous Conditions. Bull. Korean Chem. Soc..

[83] Ghodasara H.B., Vaghasiya R.G., Patel B.G., Shah V.H. (2014). Synthesis of Novel and Highly Functionalized Pyrimidine-5-carboxylate Derivatives and their Antimicrobial Evaluation. Lett. Drug Des. Discov..

[84] Meenakshi C., Ramamoorthy Y., Muthusubramanian S., Sivasubramanian S. (2001). Microvawe Assisted Synthesis of 4,6-Diarylpyridazin-3(2*H*)-ones in Solid State. Synth. Commun..

[85] Abd Rabo Moustafa M.M., Pagenkopf B.L. (2010). Synthesis of 5-Azaindoles via a Cycloaddition Reaction between Nitriles and Donor-Acceptor Cyclopropanes. Org. Lett..

[86] Ghosh P., Das J., Sarkar A., Ng S.W., Tiekink E.R.T. (2012). Oxidation with selenium dioxide: The first report of solvent-selective steroidal aromatization, efficient access to 4b,7a-dihydroxy steroids. Tetrahedron.

[87] Ghosh A., Saha B., Pradhan B.P., Ghosh P. (2013). Selenium dioxide oxidation of oxime derivative of lupanone and antimicrobial activity of the oxidized products. Res. J. Chem. Sci..

[88] Yalgudre R.S., Gokavi G.S. (2012). Selenium dioxide catalysed oxidation of acetic acid hydrazide by bromate in aqueous hydrochloric acid medium. J. Chem. Sci..

[89] Yalgudre R.S., Gokavi G.S. (2013). Kinetics and mechanism of uncatalyzed and selenium dioxide catalyzed oxidation of nicotinic acid hydrazide by bromate. Indian J. Chem. Technol..

[90] Młochowski J., Giurg M. (2009). New Trends in Chemistry and Application of Aromatic and Related Selenaheterocycles. Top. Heterocycl. Chem..

[91] Yamazaki S., Katritzky A., Ramsden C., Scriven E.F.V., Taylor R.J.K. (2008). Three or four Heteroatoms Including at Least One Selenium or Tellurium. Comprehensive Heterocyclic Chemistry III.

[92] Młochowski J., Kloc K., Lisiak R., Potaczek P., Wójtowicz H. (2007). Developments in the chemistry of selenaheterocyclic compounds of practical importance in synthesis and medicinal biology. ARKIVOC.

[93] Al-Smadi M., Ratrout S. (2004). New 1,2,3-Selenadiazole and 1,2,3-Thiadiazole Derivatives. Molecules.

[94] Al-Smadi M., Al-Momani F. (2008). Synthesis, Characterization and Antimicrobial Activity of New 1,2,3-Selenadiazoles. Molecules.

[95] Selvam C., Nidhin P., Shanmugam M., Paramasivam M., Perumal M., Dharmarajan S. (2011). A facile synthesis of carbocycle-fused mono and bis-1,2,3-selenadiazoles and their antimicrobial and antimycobacterial studies. Eur. J. Med. Chem..

[96] El-Desoky E.I., Badria F.A., Abozeid M.A., Kandeel E.A., Abdel-Rahman A.H. (2013). Synthesis and antitumor studies of novel benzopyrano-1,2,3-selenadiazole and spiro[benzopyrano]-1,3,4- thiadiazoline derivatives. Med. Chem. Res..

[97] Prabakaran K., Khan F.R.N., Jin J.S., Jeong E.D., Manivel P. (2011). Facile synthesis of 3- aryl-1- ((4-aryl-1,2,3-selenadiazol-5-yl)sulfanyl)isoquinolines. Chem. Pap..

[98] Saravanan S., Amuthavalli A., Muthusubramanian S. (2009). Synthesis and characterization of 5-(2-nitro-1-arylpropyl)-4-aryl-1,2,3-selenadiazoles. Indian J. Chem. Sect. B.

[99] Zhan P., Liu X., Fang Z., Pannecouque C., de Clercq E. (2009). 1,2,3-Selenadiazole tioacetanilides: Synthesis and anti-HIV activity evaluation. Bioorg. Med. Chem..

[100] Padmavathi V., Mahesh K., Subbaiah D.R.C.V., Padmaja A. (2008). A new class of s ulfur-linked bis-1,2,3-selenadiazoles, 1,2,3-thiadiazoles, and 2*H*-diazaphospholes. Heteroatom Chem..

[101] Aitken R.A. (2004). Product class 27: Selenazoles and tellurazoles containing one or more other heteroatoms. Sci. Synth..

[102] Grivas R. (2004). 2,1,3-Benzoselenadiazoles as Valuable Synthetic Intermediates. Curr. Org. Chem..

[103] Suzuki T., Tsui F., Okubo T., Okada A., Obana Y., Fukushima T., Miyashi T. (2001). Preparation, Structure, and Amphoteric Redox Properties of *p*-Phenylenediamine-type Dyes Fused with a Chalcogenadiazole Unit. J. Org. Chem..

[104] Kumar V., Anand A., Mahajan M.P. (2006). SeO_2_-Mediated Oxidation of 1,3-Diazabuta-1,3-dienes: A Single-Pot Synthesis of Functionalized 4-Hydroxyimidazoles. Synlett.

[105] Shaw A.Y., Denning C.R., Hulme C. (2013). One-pot two-step synthesis of quinoxalinones and diazepinones via a tandem oxidative amidation-deprotection-cyclization sequence. Synthesis.

[106] Zhang Y., Tanimoto H., Nishiyama Y., Morimoto T., Kakiuchi K. (2011). Synthesis of hetarenoindanone based on selenium dioxide-promoted direct intramolecular cyclization. Heterocycles.

[107] Ló S.M.S., Ducatti D.R.B., Duarte M.E.R., Barreira S.M.W., Noseda M.D., Gonçalves A.G. (2011). Synthesis of meso-tetraarylporphyrins using SeO_2_ as oxidant. Tetrahedron Lett..

[108] Kim H.S., Jeong K. (1999). Synthesis of Novel 1,2-Diazepino[3,4-*b*]quinoxaline and Pyridazino[3,4-*b*]quinoxaline Derivatives. J. Korean Chem. Soc..

[109] Takami S., Oshida A., Tawada Y., Kashino S., Satake K., Kimura M. (2000). Selenium Dioxide Oxidations of Dialkyl-3*H*-Azepines: The First Synthesis of 2-Azatropone from Oxidation of 2,5-Di-tert-butyl-3*H*-azepine. J. Org. Chem..

[110] Brząszcz M., Maposah M., Kloc K., Młochowski J. (2000). Selenium(IV) oxide catalyzed oxidation of aldehydes to carboxylic acids with hydrogen peroxide. Synth. Commun..

[111] Shah K.J., Chandla S.B. (1993). Liquid-phase oxidation accompanied by skeletal rearrangement of acetophenone and acetone by hydrogen peroxide in the presence of selenium dioxide. J. Chem. Technol. Biotechnol..

[112] Rohman M.R., Kharkongor I., Rajbangshi M., Mecadon H., Laloo B.M., Sahu P.R., Kharbangar I., Myrboh B. (2012). One-Pot Synthesis of Unsymmetrical Benzils by Oxidative Coupling Using Selenium Dioxide and *p*-Toluenesulfonic Acid Monohydrate. Eur. J. Org. Chem..

[113] Levchenko K.S., Semenova I.S., Yarovenko V.N., Shmelin P.S., Krayushkin M.M. (2012). Facile syntheses of 2-substituted 3-cyanochromones. Tetrahedron Lett..

[114] Vollbrecht S., Dobreva G., Cartis I., du Mont W.W., Jeske J., Ruthe F., Jones P.G., Ernst L., Grahn W., Papke U. (2008). Building the “phosphoindigo” backbone by oxidative coupling of phosphindolin-3-ones with selenium dioxide. Z. Anorg. Allg. Chem..

[115] Shaw A.Y., Denning C.R., Hulme C. (2012). Selenium dioxide-mediated synthesis of α-ketoamides from arylglyoxals and secondary amines. Tetrahedron Lett..

[116] Priewisch B., Rück-Braun K. (2005). Preparation of nitrosoarenes for the synthesis of azobenzenes. J. Org. Chem..

[117] Pansare S.V., Malusare M.G. (1997). Oxidation of 1,2,5-Thiadiazolidine 1,1-Dioxides: Synthesis of Diaryl 1,2-Diketones. Synlett.

[118] Potapov A.S., Chernova N.P., Ogorodnikov V.D., Petrenko T.V., Khlebnikov A.I. (2011). Synthesisand oxidation of some azole-containing thioethers. Beilstein J. Org. Chem..

[119] Hu Y., He Q., Zhang Z., Ding N., Hu B. (2011). Oxidative desulfurization of dibenzothiophene with hydrogen peroxide catalyzed by selenium(IV)-containing peroxotungstate. Chem. Commun..

[120] Kamata K., Hirano T., Kuzuya S., Mizuno N. (2009). Hydrogen-Bond-Assisted Epoxidation of Homoallylic and Allylic Alcohols with Hydrogen Peroxide Catalyzed by Selenium-Containing Dinuclear Peroxotungstate. J. Am. Chem. Soc..

[121] Ogura F., Otsubo T., Wirth T., Ali Khan Z., Fuchs P.L. (2007). Dimethyl Selenoxide. e-EROS Encyclopedia of Reagents for Organic Synthesis.

[122] Drabowicz J., Łyżwa P., Łuczak J., Mikołajczyk M., Laur P. (1997). New procedures for the oxidation of sulphides to sulfoxides and sulfones. Phosphorus Sulfur. Silicon Relat. Elem..

[123] Goodman M.A., Detty M.R. (2006). Selenoxides as catalysts for epoxidation and Baeyer-Villiger oxidation with hydrogen peroxide. Synlett.

[124] Betzemeier B., Lhermitte F., Knochel P. (1999). A selenium catalysed epoxidation in perfluorinated solvents with hydrogen peroxide. Synlett.

[125] Ten Brink G.-J., Vis J.M., Arends I.W.C.E., Sheldon R.A. (2002). Selenium catalyzed oxidations with aqueous hydrogen peroxide. Part 3. Oxidation of carbonyl compounds under mono/bi/triphasic conditions. Tetrahedron.

[126] Back T.G., Moussa Z. (2003). Diselenides and allyl selenides as glutathione peroxidase mimetics. J. Am. Chem. Soc..

[127] Back T.G., Kuzma D., Parvez M. (2005). Aromatic derivatives and tellurium analogues of cyclic seleninate esters and spirodioxyselenuranes that act as glutathione peroxidase mimetics. J. Org. Chem..

[128] Press D.J., Mercier E.A., Kuzma D., Back T.G. (2008). Substituent effects upon the catalytic activity of aromatic cyclic seleninate esters and spirodioxyselenuranes that act as glutathione peroxidase mimetics. J. Org. Chem..

[129] Press D.J., McNeil N.M.R., Hambrook M., Back T.G. (2014). Effects of Methoxy Substituents on the Glutathione Peroxidase-like Activity of Cyclic Seleninate Esters. J. Org. Chem..

[130] Nascimento V., Alberto E.E., Tondo D.W., Dambrowski D., Detty M.R., Nome F., Braga A.L. (2012). GPx-Like Activity of Selenides and Selenoxides: Experimental Evidence for the Involvement of Hydroxy Perhydroxy Selenane as the Active Species. J. Am. Chem. Soc..

[131] Tidei C., Piroddi M., Galli F., Santi C. (2012). Oxidation of thiols promoted by PhSeZnCl. Tetrahedron Lett..

[132] Zhang J., Koizumi T. (2000). Reactivity of Chlorooxachalcogenuranes: Oxidation of Sulfides to Sulfoxides Using Chlorooxaselenuranes. Synth. Commun..

[133] Francavilla G., Drake M.D., Bright F.V., Detty M.R. (2001). Dendrimeric organochalcogen catalysts for the activation of hydrogen peroxide: Improved catalytic activity through statistical effects and and cooperativity in succesive generation. J. Am. Chem. Soc..

[134] Drake M.D., Bright F.V., Detty M.R. (2003). Dendrimeric organochalcogen catalysts for the activation of hydrogen peroxide: Origins of the “dendrimeric effect” with catalysts terminating phenylseleno groups. J. Am. Chem. Soc..

[135] Bennett S.M., Tang Y., McMaster D., Bright F.V., Detty M.R. (2008). A xerogel-sequestered selenoxide catalyst for bromination with hydrogen peroxide and sodium bromide in an aqueous environment. J. Org. Chem..

[136] Back T.G., Fuchs P.L. (2001). Benzeneseleninic acid. e-EROS Encyclopedia of Reagents for Organic Synthesis.

[137] Renga J.M., Fuchs P.L. (2001). *o*-Nitrobenzeneseleninic Acid. e-EROS Encyclopedia of Reagents for Organic Synthesis.

[138] Renga J.M., Fuchs P.L. (2001). 2,4-Dinitrobenzeneseleninic Acid. e-EROS Encyclopedia of Reagents for Organic Synthesis.

[139] Ninomiya I., Hashimoto C., Kiguchi T., Naito T., Barton D.H.R., Luchinchi X., Milliet P. (1990). Dehydrogenation with Benzeneseleninic Anhydride in Total Synthesis of Ergot Alkaloids. J. Chem. Soc. Perkin. Trans. 1.

[140] Syper L., Mlochowski J. (1984). A Covenient Oxidation of Halomethylarenes and Alcohols to Aldehydes with Dimethyl Selenoxide and Potassium Seleninate. Synthesis.

[141] Crich D., Zou Y. (2004). Catalytic allylic oxidation with a recyclable, fluorous seleninic acid. Org. Lett..

[142] Crich D., Zou Y. (2005). Catalytic oxidation adjacent to carbonyl groups and at benzylic positions with a fluorous seleninic acid in the presence of iodoxybenzene. J. Org. Chem..

[143] Sheng S.R., Huang X. (2003). Oxidative conversion of aldoximes into carboxylic acid esters catalysed by polystyrene-bound phenylseleninic acid. J. Chem. Res..

[144] Drake M.D., Bateman M.A., Detty M.R. (2003). Substituent effects in arylseleninic acid-catalyzed bromination of organic substrates with sodium bromide and hydrogen peroxide. Organometallics.

[145] Goodmanm M.A., Detty M.R. (2004). Selenoxides as catalysts for the activation of hydrogen peroxide. Bromination of organic substrates with sodium bromide and hydrogen peroxide. Organometallics.

[146] Hopf H., Hucker J., Ernst L. (2007). On the functionalization of [2.2](1,4)phenanthrenoparacyclophane. Eur. J. Org. Chem..

[147] Dutta D., Datta A., vander Velde D.G., Georg G.I. (2007). Oxidation of baccatin III at C14. A facile rearrangement of the baccatin III core. Lett. Org. Chem..

[148] Boyer J., Bernardes-Génisson V., Nepveu F. (2003). Access to unsymmetrical 1,2-diketone immediates via benzeneseleninic anhydride-promoted oxidation: Application to indolone-*N*-oxide synthesis. J. Chem. Res. Synop..

[149] Toki N., Satoh T. (2004). Selective oxidation of alcohols at the benzylic position by benzeneseleninic anhydride. Chem. Pharm. Bull..

[150] Koepke T., Pink M., Zaleski J.M. (2006). Elucidation of the extraordinary 4-membered pyrrole ring-contracted azeteoporphyrinoid as an intermediate in chlorin oxidation. Chem. Commun..

[151] Jastrzebska I., Dobrogowska A., Lutostanska E., Morzycki J.W. (2010). On reactions of spirostane sapogenins with benzeneseleninic anhydride. Tetrahedron.

[152] Mercier E.A., Smith C.D., Parvez M., Back T.G. (2012). Cyclic Seleninate Esters as Catalysts for the Oxidation of Sulfides to Sulfoxides, Epoxidation of Alkenes, and Conversion of Enamines to α-Hydroxyketones. J. Org. Chem..

[153] Byers J.H., Nguyen T., Kobayashi Y., Fuchs P.L. (2007). Diphenyl Diselenide. e-EROS Encyclopedia of Reagents for Organic Synthesis.

[154] Ścianowski J., Rafinski Z., Wojtczak A. (2006). Syntheses and reactions of new optically active terpene dialkyl diselenides. Eur. J. Org. Chem..

[155] Syper L., Młochowski J. (1987). Benzeneperoxyseleninic acids—Synthesis and properties. Tetrahedron.

[156] Van der Toorn J.C., Kemperman G., Sheldon R.A., Arends I.W.C.E. (2009). Diphenyldiselenide- Catalyzed Selective Oxidation of Activated Alcohols with *tert*-Butyl Hydroperoxide: New Mechanistic Insights. J. Org. Chem..

[157] Giurg M., Said S.B., Syper L., Młochowski J. (2001). One-pot oxidation of azomethine compounds into arene carboxylic acids. Synth. Commun..

[158] Giurg M., Młochowski J., Ambrożak A. (2002). Hydrogen peroxide oxidation of *N,N*- dimethylhydrazones promoted by selenium compounds, titanosilicalites or acetonitrile. Polish. J. Chem..

[159] Giurg M., Młochowski J. (1999). Oxidative ring contraction of cycloalkanones: A facile method for synthesis of medium ring cycloalkane carboxylic acids. Synth. Commun. 1.

[160] Giurg M., Kowal E., Muchalski H., Syper L., Młochowski J. (2009). Catalytic oxidative domino degradation of alkyl phenols towards 2-and 3-substituted muconolactones. Synth. Commun..

[161] Giurg M., Muchalski H., Kowal E. (2012). Oxofunctionalized Trans-2-carboxycinnamic Acids by Catalytic Domino Oxidation of Naphthols and Hydronaphthoquinones. Synth. Commun..

[162] Ten Brink G.J., Martijn J.M., Vis J.H., Arends I.W.C.E., Sheldon R.A. (2001). Selenium-catalyzed oxidations with aqueous hydrogen peroxide. 2. Baeyer-Villiger reactions in homogenous solution. J. Org. Chem..

[163] Ten Brink G.-J., Fernandes B.C.M., van Vliet M.C.A., Arends I.W.C.E., Sheldon R.A. (2001). Selenium catalyzed oxidations with aqueous hydrogen peroxide. Part I. Epoxidation reactions in homogenous solution. J. Chem. Soc. Perkin Trans. 1.

[164] Zhang X., Ye J., Yu L., Shi X., Zhang M., Xu Q., Lauters M. (2015). Organoselenium-Catalyzed Baeyer-Villiger Oxidation of α,β-Unsaturated Ketones by Hydrogen Peroxide to Access Vinyl Esters. Adv. Synth. Catal..

[165] De Torres M., Arends I.W.C.E., Mayoral J.A., Pires E., Jimenez-Oses G. (2012). A highly efficient green and recoverable catalytic system for the epoxidation of fatty esters and biodiesel with H_2_O_2_ Appl. Catal. A: General.

[166] Ichikawa H., Usami Y., Arimoto M. (2005). Synthesis of novel organoselenium as catalyst for Bayer-Villiger oxidation with 30% H_2_O_2_. Tetrahedron Lett..

[167] Yu L., Wang J., Chen T., Wang Y., Xu Q. (2014). Recyclable 1,2-bis[3,5-bis(trifluoromethyl)phenyl]diselane-catalyzed oxidation of cyclohexene with H_2_O_2_: A practical access to *trans*-1,2-cyclohexanediol. Appl. Organomet. Chem..

[168] Miyake Y., Nishibayashi Y., Uemura S. (2002). Asymmetric Baeyer-Villiger Oxidation of Cyclic Ketones Using Chiral Organoselenium Catalysts. Bull. Chem. Soc. Jpn..

[169] Orentas E., Bagdziunas G., Berg U., Zilinskas A., Butkus E. (2007). Enantiospecific synthesis and chiroptical properties of bicyclic enones. Eur. J. Org. Chem..

[170] Yu L., Li H., Zhang X., Ye J., Liu J., Xu Q., Lautens M. (2014). Organoselenium-Catalyzed Mild Dehydration of Aldoximes an Unexpected Practical Method for Organonitrile Synthesis. Org. Lett..

[171] Brodsky B.H., Du Bois J. (2005). Oxaziridine-Mediated Catalytic Hydroxylation of Unactivated 3 °C-H Bonds Using Hydrogen Peroxide. J. Am. Chem. Soc..

[172] Santoro S., Santi C., Sabatini M., Testaferi L., Tiecco M. (2008). Eco-Friendly olefin dihydroxylation catalyzed by diphenyl diselenide. Adv. Synth. Catal..

[173] Zhao D., Johansson M., Bäeckvall J.E. (2007). *In situ* generation of nitroso compounds from catalytic hydrogen peroxide oxidation of primary aromatic amines and their one-pot use in hetero-Diels-Alder reactions. Eur. J. Org. Chem..

[174] Alberto E.E., Braga A.L., Detty M.R. (2012). Imidazolium-containing diselenides for catalytic oxidations with hydrogen peroxide and sodium bromide in aqueous solutions. Tetrahedron.

[175] Mugesh G., Singh H.B. (2000). Synthetic organoselenium compounds as antioxidants: Glutathione peroxidase activity. Chem. Soc. Rev..

[176] Mugesh G., Du Mont W.W., Sies H. (2001). Chemistry of Biologically Important Synthetic Organoselenium Compounds. Chem. Rev..

[177] Soriano-Garcia M. (2004). Organoselenium Compounds as Potential Therapeutic and Chemopreventive Agents: A Review. Curr. Med. Chem..

[178] Antony S., Bayse C.A. (2011). Modeling the Mechanism of the Glutathione Peroxidase Mimic Ebselen. Inorg. Chem..

[179] Bhabak K.P., Mugesh G. (2010). Functional Mimics of Glutathione Peroxidase: Bioinspired Synthetic Antioxidants. Acc. Chem. Res..

[180] Giurg M., Wiech E., Piekielska K., Gębala M., Młochowski J., Wolañski M., Ditkowski P., Peczyńska-Czoch W. (2006). A New Approach to Synthesis of Questiomycin A: Oxidative Cyclocondensation of *o*-Aminophenol. Pol. J. Chem..

[181] Giurg M., Piekielska K., Gębala M., Ditkowski P., Wolański M., Peczyńska-Czoch, Młochowski J. (2007). Catalytic Oxidative Cyclocondensation of *o*-Aminophenols to 2-Amino-3*H*- phenoxazin-3-ones. Synth. Commun..

[182] Wójtowicz H., Brząszcz M., Kloc K., Młochowski J. (2001). Selective oxidation of aromatic aldehydes to arenecarboxylic acids using ebselen-*tert*-butyl hydroperoxide catalytic system. Tetrahedron.

[183] Sarma B., Mugesh G. (2008). Antioxidant Activity of the Anti-Inflammatory Compound Ebselen: A Reversible Cyclization Pathway via Selenenic and Seleninic Acid Intermediates. Chem. -Eur. J..

[184] Giurg M., Wójtowicz H., Młochowski J. (2002). Hydroperoxide oxidation of azomethines and alkylarenes catalyzed by ebselen. Pol. J. Chem..

[185] Wójtowicz H., Młochowski J., Syper L., Yadav H.S. (2006). *t*-Butyl hydroperoxide oxidative dealkylation of hydroquinone ethers to 1,4-quinones. Synth. Commun..

[186] Balkrishna S.J., Prasad C.D., Panini P., Detty M., Chopra D., Kumar S. (2012). Isoselenazolones As Catalysts for the Activation of Bromine: Bromolactonization of Alkenoic Acids and Oxidation of Alcohols. J. Org. Chem..

[187] Wójtowicz-Młochowska H., Soroko G., Młochowski J. (2008). New recoverable organoselenium catalyst for hydroperoxide oxidation.of organic substrates. Synth. Commun..

[188] Giurg M., Brząszcz M., Młochowski J. (2006). Hydroperoxide oxidation of different organic compounds catalyzed by silica-supported selenenamide. Pol. J. Chem..

[189] Młochowski J., Kloc K., Syper L., Inglot A.D., Piasecki E. (1993). Aromatic and azaaromatic diselenides, benzisoselenazolones and related compounds active in humans: Synthesis and properties. Liebigs Ann. Chem..

[190] Pfeiffer W.D. (2001). Annulated selenazole compounds. Sci. Synth..

[191] Młochowski J., Katritzky A., Ramsden C., Scriven E.F.V., Taylor R.J.K. (2008). 1,2-Selenazoles. Comprehensive Heterocyclic Chemistry III.

[192] Zade S.S., Panda S., Singh H.B., Wolmershaüser G. (2005). Synthesis of diaryl selenides using the *in situ* reagent SeCl_2_. Tetrahedron Lett..

